# Vinylene-linked diketopyrrolopyrrole chromophores for electrochromism†

**DOI:** 10.1039/d4ra01280a

**Published:** 2024-03-26

**Authors:** Vinutha K. V., Rajeswara Rao M.

**Affiliations:** a Department of Chemistry, IIT Dharwad, WALMI Campus Dharwad 580011 Karnataka India rajesh@iitdh.ac.in

## Abstract

We report a novel series of vinylene-linked DPP compounds (1–5) formed *via* Knoevenagel condensation of dimethyl DPP (6) with various aromatic aldehydes. Incorporating the vinylene linkage and photo- and electro-active groups offered the distinct advantage of extending π-delocalization, resulting in deep-coloured solids with absorption maxima extending to 620–680 nm and low redox potentials. The DPP-triphenylamine compound (5) showed electrochromism in the near-infrared region. The colour of the solution changed from blue to green, and the absorption from 680 nm to 740 nm, leading to NIR-to-NIR absorption switching. This system exhibits rapid switching, swift response times (1.4 s and 1.2 s), and reversibility in electrochromic behaviour.

## Abstract 2

Novel series of vinylene-linked DPP compounds were synthesized *via* Knoevenagel condensation of dimethyl DPP (6) with various aromatic aldehydes. The compounds are redox active and exhibit reversible electrochromism with NIR-to-NIR switching.

## Graphical abstract

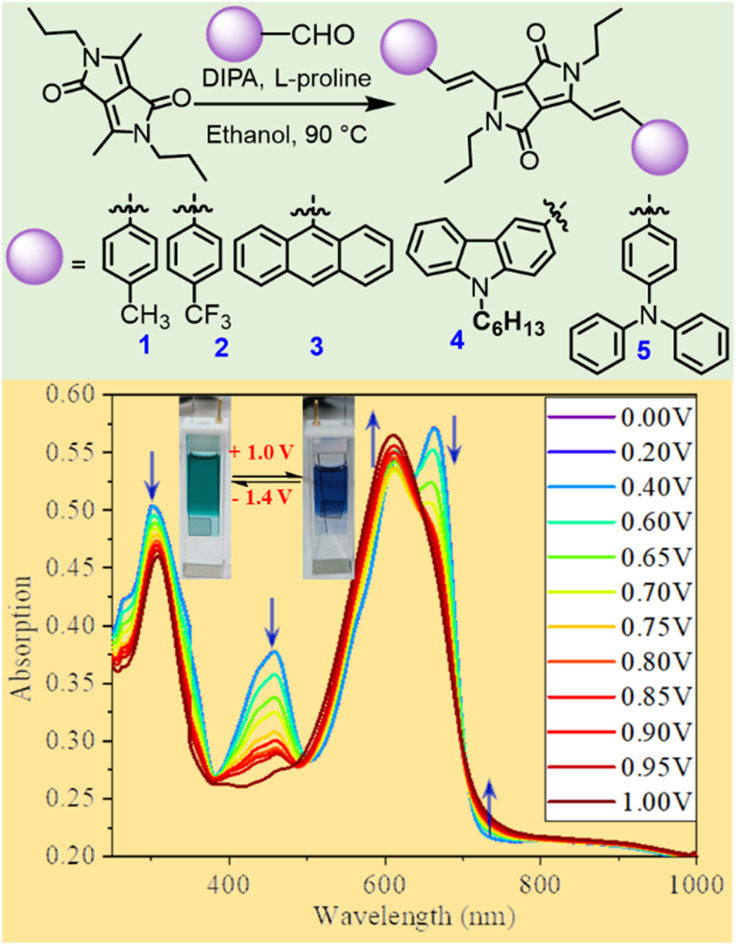

## Introduction

Diketopyrrolopyrrole (DPP), due to its special optical and electrical characteristics, has garnered significant attention as an electron-accepting π-conjugated compound. These attributes include robust visible light absorption and emission, exceptional fluorescence quantum yields, intense colouration, and remarkable charge mobility.^1–8^ Furthermore, DPP exhibits excellent thermal and photostability and can be readily functionalized, making it a versatile platform for developing various DPP-based small molecules and polymers for a wide array of applications such as organic solar cells, organic field-effect transistors (OFETs), and sensors.^9–16^

Given the popularity of DPP systems, diverse synthetic approaches have been devised to fine-tune the π-structure and properties of DPP, leveraging groups like –NH, –C=O, and aromatic units.^7,17–20^ While a few examples of DPP systems undergoing structural modifications through –NH and –C=O transformations have been reported, these examples remain limited in number. Typically, the most common approach to alter the DPP core involves varying the aryl substituents or conducting reactions on these substituents.^21,22^ However, aryl units often lead to steric hindrance-induced twisting, thus limiting π-delocalization. Consequently, heteroaromatic substituents like thiophene are frequently employed as aryl substituents to enhance planarization and π-delocalization between the DPP and aryl groups.^23^ Another approach to improve π-delocalization is introducing quinoidalization in the molecular backbone, which flattens the DPP and aromatic core by incorporating a vinylene linkage between them.^24,25^ While this strategy enables efficient electron delocalization, it compromises some essential properties associated with aromatic systems, such as fluorescence and thermodynamic stability. To overcome the above-mentioned limitation and to boost π-delocalization, Colleen N. Scott *et al.* recently developed novel DPP systems possessing vinylene linkages between the DPP core and the substituted aryl units.^26^ The compounds have been achieved *via* Knoevenagel condensation of 3,5-dimethyl DPP building block and various aromatic aldehydes. Directly flanking the DPP core with vinylene groups enabled extended π-delocalization and shifted the absorption into the near-infrared (NIR) region. For instance, the vinyl phenyl DPP exhibited an absorption peak at 600 nm, compared to 470 nm for the phenyl-linked DPP.^27^ Later, polymers developed using these vinylene-flanked DPPs displayed an absorption edge extending to 1000 nm and showcased high hole mobility (0.25 cm^2^ V^−1^ s^−1^) and photodetector efficiency in the NIR region (103 A W^−1^ at 800 nm and 102 A W^−1^ at 1050 nm).^24,28^ Building on this innovative structural approach, we coupled the DPP core with photo- and electro-active building block derivatives and investigated their optical and electrochromic behaviour.

Electrochromic materials exhibit different optical properties during an electrochemical reaction. Owing to their unique properties, such as low working voltage and high colour contrast, these materials have been found to have applications in smart windows, anti-glare rearview mirrors of vehicles, e-paper, sunglasses, molecular logic gates, memory devices, *etc.*^29^ Although metal oxide-based electrochromic (WO_3_, MoO_3_, V_2_O_5_) materials have been widely developed for electrochromic applications,^30,31^ the organic counterparts offer distinct advantages, including rapid switching speeds, excellent colouration efficiency, and easy processability.^32,33^ Most importantly, their ease of structural tunability enables precise control over colour tuning. Consequently, researchers have successfully developed numerous electronic materials, small molecules and polymers to exploit these characteristics. The electroactive building units used in organic electrochromic materials are triphenylamine,^34,35^ thienoisoindigo,^36^ phenothiazine,^37^ tetraphenyldiamine,^38^ viologen,^39^ napthalenediimide,^40^ benzodipyrrolidine^41^ including DPP.^42^ However, most electrochromic compounds developed using these building units absorb in the visible region. Thus, electrochromic switching of visible-to-visible or visible-to-NIR has been commonly observed, while NIR-to-NIR switching is infrequent. NIR electrochromic materials possess the special advantages of energy-saving windows that block the NIR light of solar irradiation and optical attenuators in telecommunications.^31,43^

In this work, we synthesized a series of vinylene-linked DPP compounds featuring electroactive groups like anthracene, carbazole, and triphenylamine. The incorporation of the vinylene linkage offers the distinct advantage of extending π-delocalization, resulting in the absorption of these compounds in the near-infrared (NIR) region. Particularly, the system containing triphenylamine demonstrates notable NIR-to-NIR switching electrochromism, enabling colour transitions from blue to green. This system exhibits rapid switching, swift response times (1.4 s and 1.2 s), and remarkable reversibility (till 48 cycles) in its electrochromic behaviour.

## Results and discussions

**Scheme 1:**
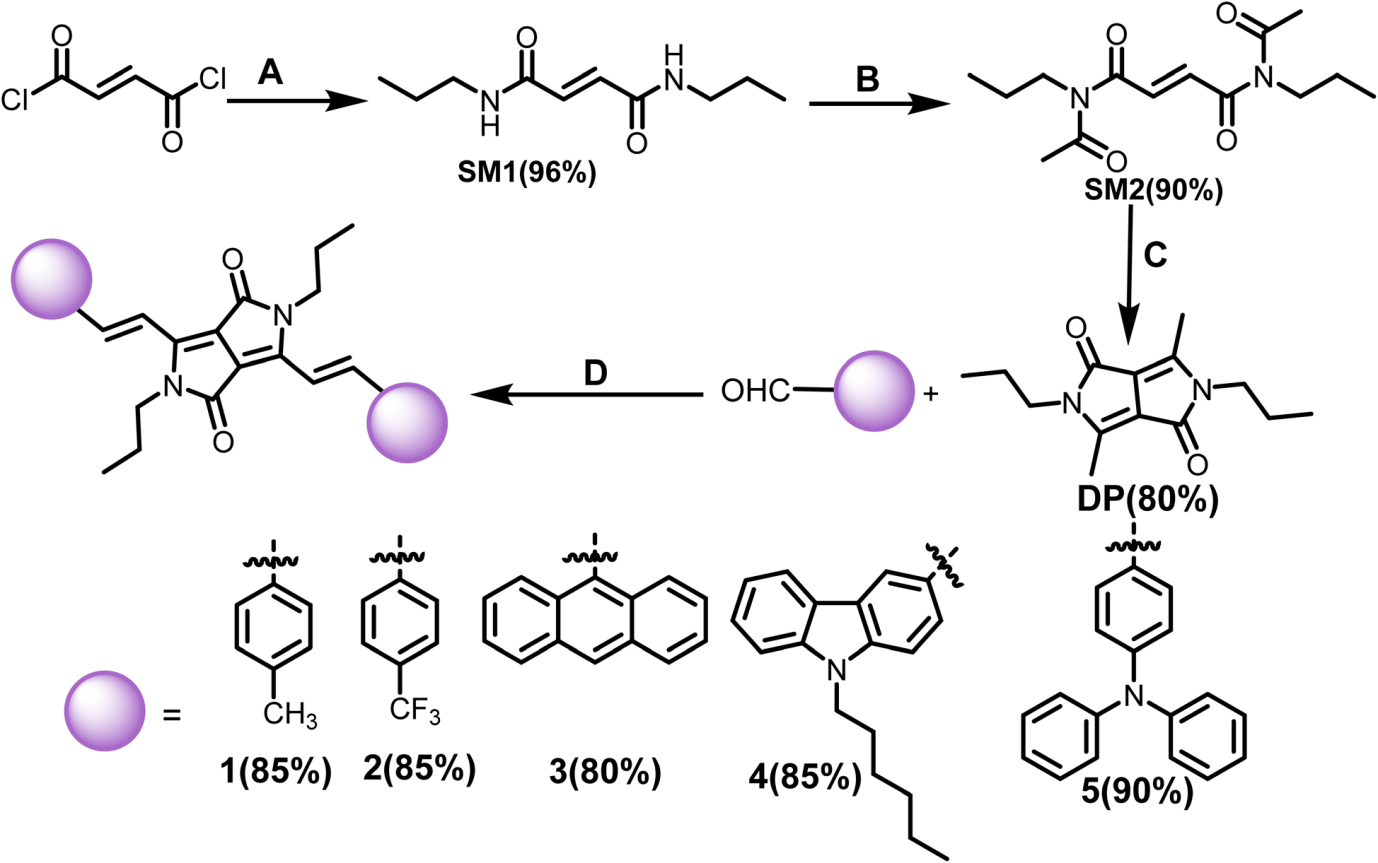
Synthesis of compounds 1–5*via* Knoevenagel condensation ((A) *n*-propylamine, NEt_3_, ACN, 0 °C to rt, 4 h; (B) DMF, isopropenylacetate, *p*-toluenesulfonicacid, reflux, 16 h; (C) pyridiniumparatolenesulfonate, PPh_3_, DMF, reflux 12 h; (D) l-proline, ethanol, DIPA, 100 °C, 12 h).

Vinylene-linked DPP-based molecules (1–5) have been developed following an established synthetic route in several steps.^26^ The key precursor, 3,6-dimethyl-2,5-dihydropyrrolo[3,4-*c*]pyrrole-1,4-dione (DP), was synthesized by converting fumaryl chloride to fumaramide (SM1) followed by *N*-acylation (SM2) and intramolecular cyclization. Subsequently, Knoevenagel condensation of DP with aromatic aldehydes in the presence of diisopropyl amine (DIPA) and the catalyst, l-proline, in the ethanol solvent for 16 h at 90 °C delivered the targeted products (1–5). The compounds were isolated as dark green to purple solids in 80–90% yields (Scheme 1). 1–5 was characterized by ^1^H and ^13^C NMR spectroscopy and high-resolution mass spectrometry (HRMS). Each signal in the ^1^H NMR has been identified using coupling constants and correlated spectroscopy (COSY) and nuclear Overhauser effect spectroscopy (NOESY) spectra (Fig. S21 and S22†).

**Fig. 1:**
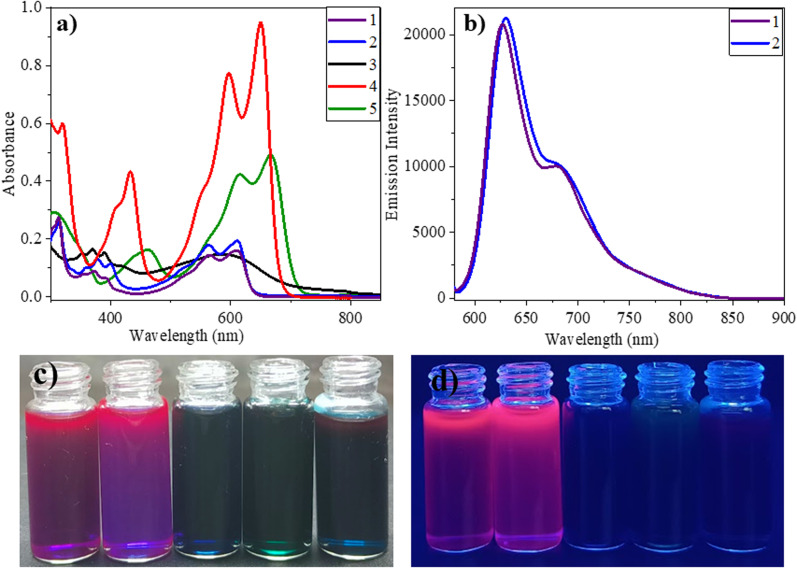
(a) Absorption spectra of compound 1–5 were measured in chloroform solvent by using 10^−5^ M concentration solution; (b) emission spectra of 1 and 2 in (*λ*_ex_ = 560 nm) measured in chloroform solution by using 10^−6^ M concentration solution; the images of 1–5 in daylight (c) and UV lamp at 365 nm (d) in chloroform solutions.

The optical properties of 1–5 were studied in a dilute chloroform solution using UV-vis absorption spectroscopy. The compounds showed two-banded absorption spectra with vibrational fine structure spanning the UV to the visible region, indicating that the vinylene bridge supports the extended π-delocalization. Compounds 1–3 exhibit a similar absorption pattern, having a high energy band appearing in the range of 325–432 nm and a low energy band around 620 nm. On the other hand, for 4 and 5, these bands significantly redshifted by 30–50 nm and appeared around 650 nm and 667 nm, respectively (Fig. 1a). This can be attributed to extended π-delocalization supported electron donating TPA/carbazole. The broad and structureless band features of 3 may be due to the intense molecular vibrations and rotations caused by the anthracenyl units. The optical band gaps of 1–3 are 2.0–2.1 eV, while they are 1.9 eV for 4 and 5. It is important to note that the solvent polarity (CHCl_3_, THF and DMF) has minimal effect on the absorption properties of the compounds (Fig. S1†).

The fluorescence properties of the compounds are studied in chloroform solution at an excitation wavelength of 560 nm. Among 1–5, compounds 1 and 2 have been found to be emissive with the emission peak centring at 630 nm and 626 nm, respectively (Fig. 1b). The extended π-delocalization within the molecules promotes the NIR-emission of the compounds. The compound's fluorescence quantum yields (*Φ*_f_) were measured to be 5.8 and 5.4%, respectively. Akin to the absorption properties, the fluorescence of the compounds also showed minimal solvent effect (Fig. S4†). The presence of π-conjugated units such as anthracene/TPA/carbazole in 3–5 promote π-delocalization and enable charge transfer interactions. Such interactions reduce the energy gap between the ground and the excited state and promote non-radiative transitions.

**Fig. 2:**
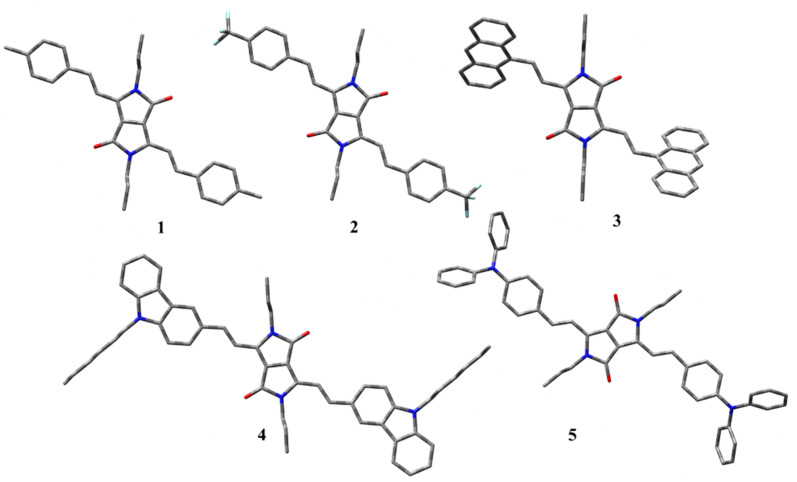
DFT geometry optimized structures of compounds 1–5.

**Fig. 3:**
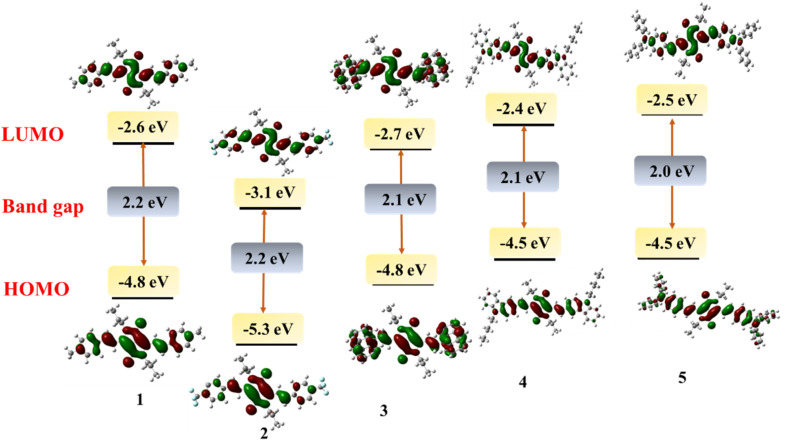
The frontier molecular orbitals (FMOs) of 1–5 computed by DFT.

To investigate the structural and electronic properties of 1–5, density functional theory (DFT) studies have been performed using the Gaussian16 program at B3LYP/6-31G(d) level basis set. The geometrically optimized structures of 1–5 present a planar conformation with the dihedral angle between the DPP unit and vinyl aryl unit to be −3.4 to −11.9° which allows the facile π-delocalization along the molecular backbone (Fig. 2). The HOMO and LUMO of 1–5 are localized on the central DPP ring extending up to the substituted aryl units. Based on the electronic effects of the substituents, the electronic distribution has varied; for example, the HOMO has distributed extensively on DPP and triphenylamine or carbazole units in 4 and 5, respectively. On the contrary, the LUMOs of these compounds are purely localized on the central DPP units (Fig. 3). A reverse trend has been noticed for 1 due to the presence of electron-withdrawing 4-(trifluoromethyl)phenyl substituent. The HOMO and LUMO energies of 1–5 were raised with an increase in the electron-donating capability of the substituents. The HOMOs of the compounds range from −4.5 eV to −5.5 eV, while the LUMOs' energy is from −3.4 eV to −2.3 eV (Table 1). The band gap of the compounds is low, indicating the extended π-delocalization, however, with a negligible variation between the compounds despite the varied electronic effects of the substituents. The absorption spectra of the compounds have also been computed *via* TD-DFT. The DFT simulated absorption spectra also displayed two electronic transitions; the high energy band is in the region of 400–480 nm while the low energy is in the range of 590–690 nm (Fig. S2†). The DFT predicted that the low energy band originates from HOMO to LUMO transition while the high energy band is due to HOMO to LUMO+1 in case of 1 and 2; LUMO+2 to HOMO, HOMO to LUMO+2, HOMO−2 to LUMO in case of 3; HOMO to LUMO+2 in case of 4; LUMO+2 to HOMO in case of 5. The peak maxima of the computed spectra deviate from the experimentally deduced values due to the gas phase calculations, but the trend is in good agreement.

**Table 1:** Electronic properties of compounds 1–5 by DFT and cyclic voltammetry method

Compound	DFT computed FMOs			Electrochemical data^a^		
	HOMO (eV)	LUMO (eV)	Band gap (eV)	Oxidation potential in V *vs.* Fc/Fc^+^ (HOMO in eV)	Reduction potential in V *vs.* Fc/Fc^+^ (LUMO in eV)	Band gap (eV)
1	−4.8	−2.6	2.2	0.5 (−5.3)	−1.7 (−3.1)	2.2
2	−5.3	−3.1	2.2	0.5 (−5.3)	−1.4 (−3.4)	1.9
3	−4.8	−2.7	2.1	0.3 (−5.1), 0.5	−1.6 (−3.2)	1.8
4	−4.5	−2.4	2.1	0.01 (−4.8), 0.3	−1.8 (−3.0)	1.8
5	−4.5	−2.5	2.0	0.1 (−4.9), 0.6	−1.8 (−3.0)	1.8

a Onset oxidation and reduction potentials.

**Fig. 4:**
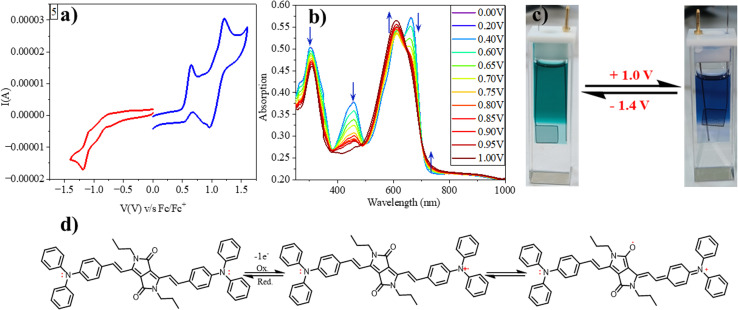
The cyclic voltammogram of compound 5 (a); spectroelectrochemical absorption spectra of compound 5 at the range of oxidation potentials varied from 0 V to +1 V at 10^−5^ M concentration (b); image showing colour change by electrochromism (c). The mechanism of reversibility of electrochromism (d).

We investigated the redox characteristics of 1–5 by employing cyclic voltammetry with a glassy carbon working electrode, a Pt wire auxiliary electrode, and an Ag/AgCl reference electrode in a 0.1 M TBAP electrolyte. We observed one oxidation peak at 0.5 V (*vs.* Fc/Fc^+^) for compound 1 corresponding to DPP, which was decreased to 0.46 V for 2 due to the electron accepting CF_3_ substituents. But for 3–5, two oxidation waves have been observed: 0.3 V/0.5 V, 0.01 V/0.3 V and 0.1 V/0.6 V, respectively. The first oxidation for 3 and second oxidation for 4, 5 have been assigned to the DPP, while the others to anthracene, carbazole and triphenylamine, respectively. As the electron donating strength of the substituents increases, the oxidation potential of the DPP unit decreases. A similar trend of increasing reduction potential upon increasing the electron-donating capability of substituents has also been observed following a numerical order of 4 (−1.8 V *vs.* Fc/Fc^+^) = 5 (−1.8 V) > 1 (−1.7 V) > 3 (−1.6 V) > 2 (−1.4 V) (Fig. 4a, S3† and Table 1). The HOMO, LUMO and bandgap values calculated using the electrochemical data align with the DFT-deduced data. The intriguing redox properties and deep colours of these vinylene-linked DPP small molecules of these compounds prompted us to delve into the electrochromic behaviour. Specifically, our focus was on a DPP molecule (5) which features a donor–acceptor–donor (D–A–D) π-structure with triphenylamine (TPA) flanking.

## Spectroelectrochemistry and electrochromism

We employed spectroelectrochemical absorption spectroscopy to study the optical changes upon applying the electric potential. At 0 V, the solution of compound 5 exhibited a green hue and displayed three absorption bands at 303 nm, 457 nm, and 662 nm. When subjected to bulk oxidation at +1 V, the solution's colour shifted to blue, with the disappearance of the 457 nm band and the appearance of new absorption 608 nm and 739 nm (Fig. 4b). This transformation is attributed to the generation of a radical cation on the TPA molecule. Subsequently, upon applying a reduction potential (−1.4 V), the blue solution reverted to its original green colour, indicating the highly reversible nature of this process (Fig. 4c).

**Fig. 5:**
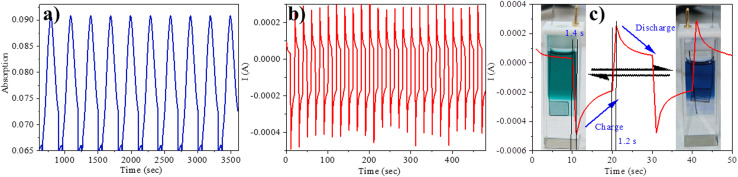
(a) Absorbance of compound 5 monitored at 610 nm *via* switching the potential between +1 V and −1.4 V *vs.* Ag/AgCl; (b) the stability of the neutral and diradical cation species of the 5*via* switching the potential between +1 V and −1.4 V *vs.* Ag/AgCl; (c) switching time of 5 electrochromism by spectroelectrochemistry.

Furthermore, we assessed the absorption spectra of compound 5 by incrementally sweeping the oxidation potential from 0 V to +1 V *vs.* Ag/AgCl. With increasing potential, there was a gradual reduction in the intensities of the absorption signals associated with the pristine compound, accompanied by the progressive emergence of near-infrared (NIR) peaks at 608 nm and 739 nm. Finally, at +1 V, the 457 nm peak completely vanished, giving way to highly intense new peaks at 608 nm and 739 nm, indicative of the complete conversion into a radical cation. The electrochemical switching of the color has been tested for 10 cycles with a residence time of 20 s at the visible range (610 nm) between 0 and 1.4 V. The switching ability of the material has been found to be stable without changing the color and any loss of absorbance (Fig. 5a). Further, the stability of the radical cation has also been tested by the current density *versus* time plot *via* multi-step chronoamperometry experiment which showed no loss of current up to 48 cycles (Fig. 5b). The switching time has been found to be 1.4 s for green to blue *via* oxidation and 1.2 s for colouring back to green *via* reduction (Fig. 5c). Overall, the high stability of the radical cation could be attributed to the DPP unit which is expected to stabilize the radical species *via* the charge delocalization (Scheme S1†).

**Fig. 6:**
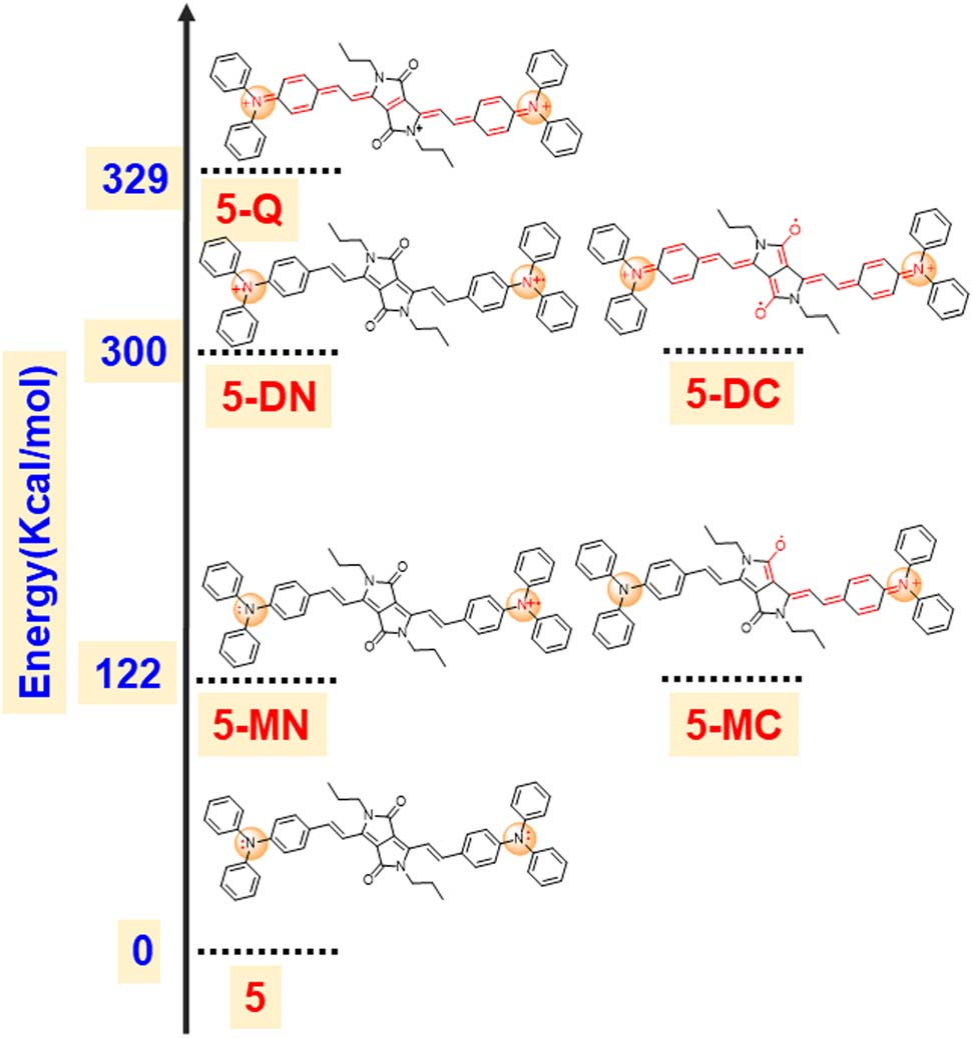
DFT computed energies of the possible intermediate species of compound 5 electrochromism.

To support our hypothesis, we optimized various radical cation species of 5 as presented in Fig. 5 and compared their energies. The calculation revealed that the mono radical cation that resides on the nitrogen of TPA (5-MN) or delocalizes between the nitrogen of TPA and carbonyl of DPP (5-MC) possesses the same energy with 122 kcal mol^−1^ higher energy compared to the neutral species. This indicates that the radical cation will delocalize between the nitrogen of TPA and the carbonyl of the DPP. Similarly, the di radical cation (5-DN) and its carbonyl counterpart carbonyl of DPP (5-DC) also possess the same energy, but its quinoidal form (5-Q) possess 29 kcal mol^−1^ of higher energy, indicating that the radical will reside on hetero atoms (either nitrogen or oxygen). Overall, the energy of diradical cations compared to neutral species is much higher (300 kcal mol^−1^), indicating that di-radical cation species are difficult to form (Fig. 6).

## Conclusion

In summary, novel vinylene linked DPP based small molecules containing anthracene, carbazole, and triphenylamine have been synthesized by Knoevenagel condensation of dimethyl DPP (6) with aromatic aldehydes in the presence of diisopropyl amine (DIPA) and the catalyst, l-proline. The compounds were isolated in 80–90% yields and characterized by ^1^H and ^13^C NMR spectroscopy and HRMS. The compounds absorb (620–680 nm) and emit in the NIR region (700 nm). The DPP-triphenylamine exhibits reversible electrochromic behaviour, changing colour from blue to green and absorption from 680 nm to 740 nm. The DPP core in the molecular framework allows the facile electron delocalization and contributes to the stability of the oxidized radical cation species. This system exhibits rapid switching and swift response times.

## Conflicts of interest

The authors declare no competing financial interest.

## Supplementary Material

RA-014-D4RA01280A-s001

## References

[cit1] Bao W. W., Li R., Dai Z. C., Tang J., Shi X., Geng J. T., Deng Z. F., Hua J. (2020). Front. Chem..

[cit2] Bürckstümmer H., Weissenstein A., Bialas D., Würthner F. (2011). J. Org. Chem..

[cit3] Pieczykolan M., Derr J. B., Chrayteh A., Koszarna B., Clark J. A., Vakuliuk O., Jacquemin D., Vullev V. I., Gryko D. T. (2021). Molecules.

[cit4] Nielsen C. B., Turbiez M., McCulloch I. (2013). Adv. Mater..

[cit5] Kaur M., Choi D. H. (2015). Chem. Soc. Rev..

[cit6] Tang A., Zhan C., Yao J., Zhou E. (2017). Adv. Mater..

[cit7] Kang S. H., Lee D., Kim H., Choi W., Oh J., Oh J. H., Yang C. (2021). ACS Appl. Mater. Interfaces.

[cit8] Bürckstümmer H., Weissenstein A., Bialas D., Würthner F. (2011). J. Org. Chem..

[cit9] Hang Y., Wang J., Jiang T., Lu N., Hua J. (2016). Anal. Chem..

[cit10] Hwang T. G., Kim J. Y., Namgoong J. W., Lee J. M., Yuk S. B., Kim S. H., Kim J. P. (2019). Photochem. Photobiol. Sci..

[cit11] Song C. E., Kim Y. J., Suranagi S. R., Kini G. P., Park S., Lee S. K., Shin W. S., Moon S. J., Kang I. N., Park C. E., Lee J. C. (2016). ACS Appl. Mater. Interfaces.

[cit12] Kim J. H., Park J. B., Yang H., Jung I. H., Yoon S. C., Kim D., Hwang D. H. (2015). ACS Appl. Mater. Interfaces.

[cit13] Wang C., Qin Y., Sun Y., Guan Y. S., Xu W., Zhu D. (2015). ACS Appl. Mater. Interfaces.

[cit14] Wang L., Lai B., Ran X., Tang H., Cao D. (2023). Molecules.

[cit15] More K. S., Mirgane H. A., Shaikh S., Perupogu V., Birajdar S. S., Puyad A. L., Bhosale S. V., Bhosale S. V. (2022). J. Org. Chem..

[cit16] Jeong Y. H., Lee C. H., Jang W. D. (2012). Chem. – Asian J..

[cit17] Sharma A., Singh R., Kini G. P., Hyeon Kim J., Parashar M., Kim M., Kumar M., Kim J. S., Lee J. J. (2021). ACS Appl. Mater. Interfaces.

[cit18] Kang S. H., Lee D., Kim H., Choi W., Oh J., Oh J. H., Yang C. (2021). ACS Appl. Mater. Interfaces.

[cit19] Frebort Š., Eliáš Z., Lyčka A., Luňák S., Vyňuchal J., Kubáč L., Hrdina R., Burgert L. (2011). Tetrahedron Lett..

[cit20] Liu S. Y., Shi M. M., Huang J. C., Jin Z. N., Hu X. L., Pan J. Y., Li H. Y., Jen A. K. Y., Chen H. Z. (2013). J. Mater. Chem. A.

[cit21] Ghosh S., Raveendran R., Saeki A., Seki S., Namboothiry M., Ajayaghosh A. (2019). ACS Appl. Mater. Interfaces.

[cit22] Hupfer M. L., Koszarna B., Ghosh S., Gryko D. T., Presselt M. (2021). Langmuir.

[cit23] Grzybowski M., Hugues V., Blanchard-Desce M., Gryko D. T. (2014). Chem. – Eur. J..

[cit24] Dhar J., Mukhopadhay T., Yaacobi-Gross N., Anthopoulos T. D., Salzner U., Swaraj S., Patil S. (2015). J. Phys. Chem. B.

[cit25] Ray S., Sharma S., Salzner U., Patil S. (2017). J. Phys. Chem. C.

[cit26] Feng D., Barton G., Scott C. N. (2019). Org. Lett..

[cit27] Dhar J., Venkatramaiah N., Anitha A., Patil S. (2014). J. Mater. Chem. C.

[cit28] Chen H., Guo Y., Yu G., Zhao Y., Zhang J., Gao D., Liu H., Liu Y. (2012). Adv. Mater..

[cit29] Gu C., Jia A. B., Zhang Y. M., Zhang S. X. A. (2022). Chem. Rev..

[cit30] Kim Y. M., Li X., Kim K. W., Kim S. H., Moon H. C. (2019). RSC Adv..

[cit31] Mortimer R. J. (1997). Chem. Soc. Rev..

[cit32] Bin Cui B., Zhong Y. W., Yao J. (2015). J. Am. Chem. Soc..

[cit33] Yan S., Zhang L., Lv X., Sun J., Zhang Y., Zhang C. (2022). Adv. Photonics Res..

[cit34] Yen H. J., Liou G. S. (2018). Polym. Chem..

[cit35] Yen H. J., Liou G. S. (2010). J. Mater. Chem..

[cit36] Yue H., Ju X., Du Y., Zhang Y., Du H., Zhao J., Zhang J. (2021). Org. Electron..

[cit37] Wu J., Zeng Z., Chen Q., Zheng J., Xu C. (2019). Opt. Mater..

[cit38] Liou G. S., Hsiao S. H., Huang N. K., Yang Y. L. (2006). Macromolecules.

[cit39] Liu H. S., Pan B. C., Huang D. C., Kung Y. R., Leu C. M., Liou G. S. (2017). NPG Asia Mater..

[cit40] Li F., Huang Z. J., Zhou Q. H., Pan M. Y., Tang Q., Bin Gong C. (2020). J. Mater. Chem. C.

[cit41] Tatemura R., Yasutake M., Kinoshita H., Miura K. (2022). J. Org. Chem..

[cit42] Cheng X., Ju X., Du H., Zhang Y., Zhao J., Xie Y. (2018). RSC Adv..

[cit43] Brooke R., Mitraka E., Sardar S., Sandberg M., Sawatdee A., Berggren M., Crispin X., Jonsson M. P. (2017). J. Mater. Chem. C.

