# Supplementary material for: Vinylene-linked diketopyrrolopyrrole chromophores for electrochromism
Source: RSC Adv. 2024 Mar 26;14(14):10017–23. doi: 10.1039/d4ra01280a (PMC10964203; doi:10.1039/d4ra01280a)
Supplement: RA-014-D4RA01280A-s001 [file RA-014-D4RA01280A-s001.pdf]

Electronic Supplementary Material (ESI) for RSC Advances.
This journal is © The Royal Society of Chemistry 2024

Supporting Information

Vinylene-linked Diketopyrrolopyrrole systems for Electrochromism

Vinutha K. V. and Rajeswara Rao M*
Department of chemistry, IIT Dharwad, Dharwad-580011, Karnataka, India
rajesh@iitdh.ac.in

Contents

List of figures:......2

1.Experimental section: 3

1.1 General Information......3

1.2 Synthetic Procedure 3

1.2.1 Synthesis of N^1,N^4-dipropylfumaramide(SM1)......3

1.2.2 Synthesis of N^1,N^4-diacetyl-N1,N4-dipropylfumaramide(SM2) 4

1.2.3 Synthesis of diketopyrrolopyrrole (DP)......4

1.2.4 General synthetic method for the compounds **1-5**. 4

2. Fabrication of working electrode:......6

3. References: 20

List of figures:

Fig. S1: Absorption solvatochromism of compounds **1-5**. 6
Fig. S 2: TD-DFT absorption spectra of compounds **1-5**. 7
Fig. S 3: Cyclic voltammogram of compounds **1**- **5** v/s Fc/Fc^{+} measured in degassed dichloromethane solvent by using tetrabutylammonium perchlorate (TBAP) as supporting electrolyte at 50 mV/s scanning rate. 7
Fig. S 4: Fluorescence solvatochromism of compounds **1-5**. 8
Fig. S 5: The Spectro electrochemical absorption spectroscopy of compound **5** reversibility test. 8
Fig. S 6: HRMS of compound **1**. 9
Fig. S 7: ^{1}H-NMR of compound **1**. 10
Fig. S 8: ^{13}C NMR of compound **1**. 10
Fig. S 9: HRMS of compound **2**. 11
Fig. S 10: ^{1}H-NMR of compound **2.** 12
Fig. S 11: ^{13}C-NMR of compound **2**. 12
Fig. S 12: HRMS of compound **3**. 13
Fig. S 13: ^{1}H NMR of compound **3**. 14
Fig. S 14: 13C-NMR of compound **3**. 14
Fig. S 15: HRMS of compound **4**. 15
Fig. S 16: ^{1}H-NMR of compound **4.** 16
Fig. S 17: ^{13}C-NMR of compound **4.** 16
Fig. S 18: HRMS of compound **5**. 17
Fig. S 19: ^{1}H-NMR of compound **5.** 18
Fig. S 20: ^{13}C-NMR of compound **5**. 18
Fig. S 21: 2D COSY spectrum of compound **5**. 19
Fig. S 22: 2D NOESY spectrum of compound **5**. 19

1.Experimental section:

1.1 General Information

All the chemicals and solvents were used as received without any further purification. The chemicals fumaryl chloride, propyl bromide, triethylamine, acetonitrile (ACN), pyridine, *p*-toluene sulfonic acid, isopropenylacetate, dimethylformamide, *p*-tolualdehyde, 4-trifluoromethylbenzaldehyde, 9-anthraldehyde, 9-hexyl-9H-carbazole-3-carbaldehyde, 4-(diphenylamino)benzaldehyde, ethanol, L-proline, di-isopropenylamine(DIPA) were purchased from Sigma-Aldrich, Tokyo Chemical Industry (TCI), Finar and Spectrochem. ^{1}H-NMR, ^{13}C-NMR, 2D COSY and 2D NOSEY were recorded in JEOL ECZS 400MHz and 100MHz respectively. UV-Vis spectra was recorded in the Cary 5000 UV-Vis NIR spectrometer. The fluorescence emission and the quantum yields were measured by using the PerkinElmer FL6500 spectrometer. Electrochemical studies were done by using Gamry INTERFACE1010 31184 in the three-electrode system by using Pt wire as a counter electrode, Ag/AgCl as a reference electrode and Glassy carbon as a working electrode. Tetrabutylammonium perchlorate (TBAP) as an electrolyte and HPLC grade dichloromethane as a solvent. Finally, the redox potentials were modified with reference to the ferrocene (Fc/Fc^{+}). The HOMOs were calculated by using the equation HOMO = -(4.8+oxd potential) and LUMOs by using LUMO = -(4.8+ Redn potential).

1.2 Synthetic Procedure

1.2.1 Synthesis of N^{1},N^{4}-dipropylfumaramide(**SM1**)[1]:

A mixture of n-propylamine (39.21 mmol, 2.32 g), triethylamine (26.14 mmol, 2.64 g) in ACN (20 mL) was taken into a RB under inert atmosphere. To which the fumarylchloride (13.07 mmol, 2g) in ACN was added dropwise over a period of 30 min at 0 ˚C. Then the reaction mixture is

stirred for 4 h more at room temperature. The obtained half white solid is filtered off and washed with excess water followed by acetone to yield SM1 in 96% yield (2.3g).

1.2.2 Synthesis of N^1,N^4-diacetyl-N1,N4-dipropylfumaramide(**SM2**)[1]

To the mixture of **SM1** (27.1 mmol, 5.38 g) and Isopropenyl acetate (542 mmol, 52.3g), 0.1 M *p*-toluenesulfonic acid in DMF (27 mL) was added. The mixture was refluxed for 16 h. The compound is extracted by using ethyl acetate and the crude product is obtained by removing solvent using rotary evaporator. The pure compound of **SM2** was obtained as orange crystals through column chromatography by using 30% of DCM in Hexane in 92% yield (7.0g).

1.2.3 Synthesis of diketopyrrolopyrrole (**DP**)[1]

The compound **SM2** (7g) in ACN was added by pyridiniumparatoluenesulfonate followed by triphenylphosphine (PPh_3) and the mixture was refluxed at 100 ˚C. The completion of the reaction is confirmed by thin layer chromatography (TLC). After the completion of the reaction the organic layer was separated and washed with an excess of water. The crude product which was obtained after evaporating the organic solid was purified through column chromatography by using 50% of DCM in hexane to yield pure compound in yellow solid in 80% yield (4.9g).

1.2.4 General synthetic method for the compounds **1**-**5**.

To the solution of DPP (0.4 mmol, 100mg) in 15 mL of ethanol was added by DIPA (1.61 mmol) followed by L-proline (0.08 mmol) and the corresponding aromatic aldehyde (0.8 mmol). The mixture was stirred at 100 ˚C for 16 h. The obtained solid is filtered off and washed with diethyl ether followed by hexane to yield the pure compounds **1**-**5**.

Compound **1** (Purple solid, Yield: 85%; 155 mg) HRMS: m/z calculated for $C_{30}H_{32}N_2O_2$ [M^+]: 452.2464; found m/z: 452.2471; ^{1}H NMR (400 MHz, $CDCl_3$) in **δ**: 8.86(2H, d, J=16.0 Hz), 7.54(4H, d, J=8.0 Hz), 7.20(4H, d, J=8.0 Hz), 6.81(2H, d, J=16.0 Hz), 3.79(4H, t, J=7.4 Hz), 2.37(6H, s), 1.72(4H, sextet, J=7.4 Hz), 1.00(6H, t, J=7.4 Hz); ^{13}C-NMR (100 MHz, $CDCl_3$) δ: 11.2, 21.4, 23.4, 42.6, 108.6, 112.7, 128.3, 130.1, 133.5, 140.6, 143.7, 144.7, 161.1.

Compound **2** (Purple solid, Yield: 85 %; 192 mg) HRMS: m/z calculated for $C_{30}H_{26}F_6N_2O_2$ [M^+]: 560.1898; found m/z: 560.1901; ^{1}H NMR (400 MHz, $CDCl_3$) in **δ**: 8.91(2H, d, J=16.0 Hz), 7.75(4H, d, J=8.2 Hz), 7.66(4H, d, J=8.2 Hz), 6.92(2H, d, J=16.0 Hz), 3.81(4H, t, J=7.3 Hz), 1.73(4H, sextet, J=7.4 Hz), 1.02(6H, t, J=7.4 Hz); ^{13}C-NMR (100 MHz, $CDCl_3$) δ: 11.6, 23.7, 41.9, 109.8, 115.5, 122.2, 126.0, 128.5, 131.4, 139.3, 141.3, 144.3, 161.6.

Compound **3** (Dark blue solid, Yield: 80%; 201 mg) HRMS: m/z calculated for $C_{32}H_{34}N_2O_2$ [M^+]: 624.2777; found m/z: 624.2763; ^{1}H NMR (400 MHz, $CDCl_3$) in **δ**: 10.02(2H, d, J=16.0 Hz), 8.61(4H, d, J=8.0 Hz), 8.50(2H, s), 8.07(4H, d, J=8.0 Hz), 7.54(4H, t, J=8.0 Hz), 7.69(4H, t, J=8.0 Hz), 7.00(2H, d, J=16.0 Hz), 3.89(4H, t, J=7.3 Hz), 1.83(4H, sextet, J=7.4 Hz), 1.06(6H, t, J=7.4 Hz); ^{13}C-NMR (100 MHz, $CDCl_3$) δ: 162.0, 146.1, 132.2, 130.0, 129.1, 128.2, 126.3, 124.1, 110.1, 42.2, 24.1, 19.1, 11.2.

Compound **4** (Dark Green solid, Yield: 85%; 264mg) HRMS: m/z calculated for $C_{52}H_{58}N_4O_2$ [M^+]: 770.4560; found m/z: 770.4554; ^{1}H NMR (400 MHz, $CDCl_3$) in **δ**: 9.16(2H, d, J=16.0 Hz), 8.42(2H, s), 8.13(2H, d, J=8.0 Hz), 7.83(2H, d, J=8.0 Hz), 7.50(2H, t, J=8.4 Hz), 7.41(4H, dd, J_1=8.0 Hz, J_2=4.0 Hz), 7.29(2H, t, J=7.7 Hz), 6.91(2H, d, J=4.0 Hz), 4.30(4H, t, J=7.3 Hz), 3.89(4H, t, J=7.3 Hz), 1.85(8H, m), 1.33(12H, m), 1.08(6H, t, J=7.3 Hz), 0.86(6H, t, J=7.2 Hz); ^{13}C-NMR

(100 MHz, $CDCl_3$) δ: 11.6, 13.7, 22.9, 24.0, 27.0, 29.0, 32.3, 42.3, 43.3, 108.5, 109.3, 111.1, 119.8, 120.7, 122.2, 123.0, 123.9, 125.7, 126.0, 128.1, 140.6, 142, 144.2, 144.9, 161.8.

Compound **5** (Dark Purple solid, Yield: 90%; 275mg) HRMS: m/z calculated for $C_{52}H_{46}N_4O_2$ [M^+]: 758.3621 ; found m/z: 758.3605; ^{1}H NMR (400 MHz, $CDCl_3$) in **δ**: 8.81(2H, d, J=16.0 Hz), 7.49(4H, d, J=8.0 Hz), 7.28(8H, t, J=6.8 Hz), 7.11(12H, m), 6.05(4H, d, J=8.0 Hz), 6.70(2H, d, J=16.0 Hz), 3.76(4H, t, J=7.4 Hz), 1.71(4H, sextet, J=7.5 Hz), 0.98(6H, t, J=7.4 Hz); ^{13}C-NMR (100 MHz, $CDCl_3$) δ: 11.7, 23.5, 42.2, 108.6, 111.3, 122.0, 124.1, 125.2, 129.3, 142.6, 144.3, 147.2, 147.3, 149.8, 162.4.

2. Fabrication of working electrode:

The gel was made through the amalgamation of the compound and TBAP, utilizing a 1:100 ratio in dichloromethane (DCM) solvent along with polymethylmethacrylate (PMMA) as a binding agent. The blend was vigorously stirred until a uniformly mixed gel was achieved. Subsequently, the gel was evenly applied to the ITO electrode and thoroughly dried.

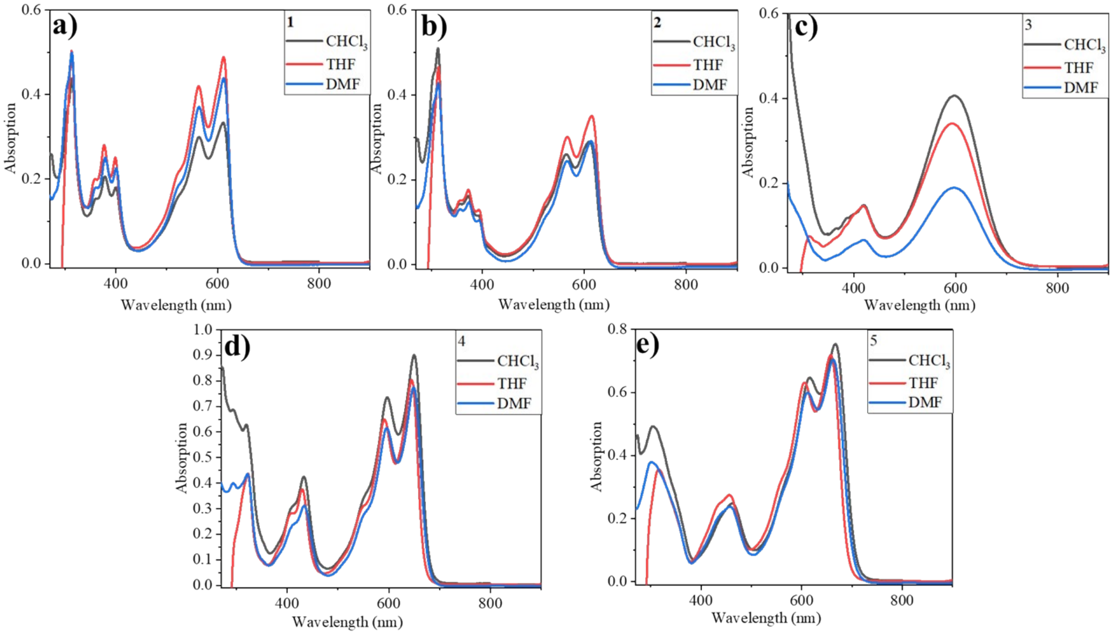

Fig. S 1: Absorption solvatochromism of compounds **1**-**5**.

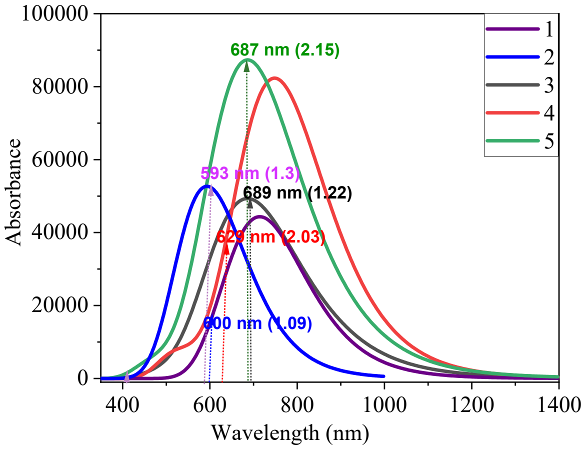

Fig. S 2: TD-DFT absorption spectra of compounds **1-5**.

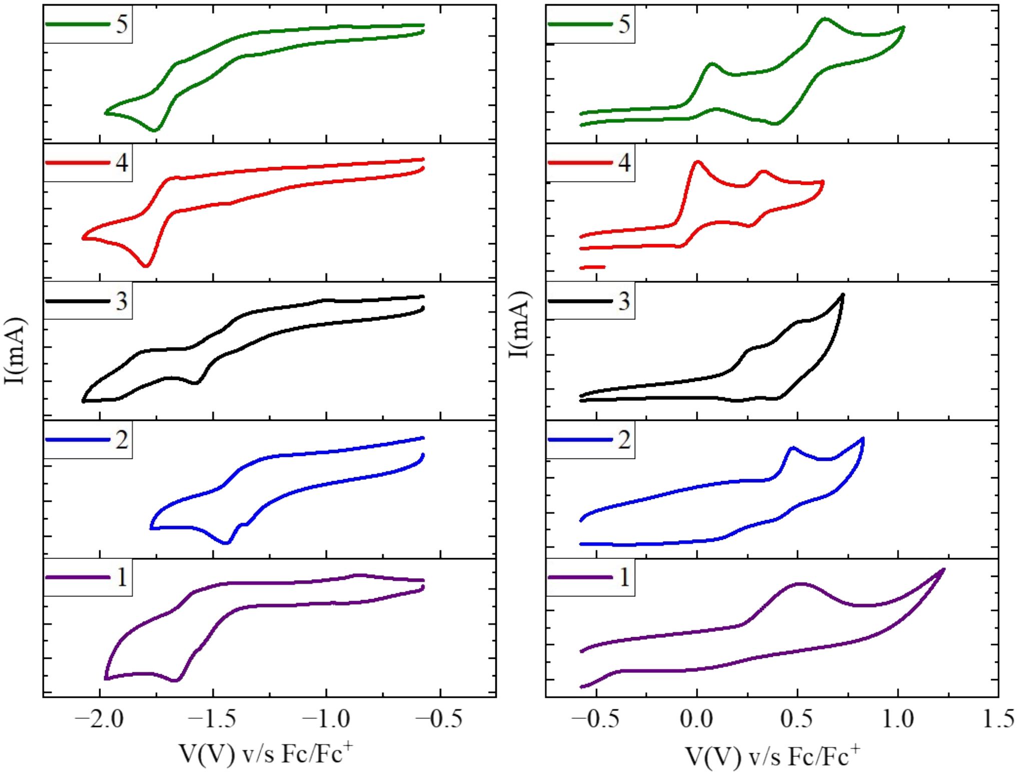

Fig S 3: Cyclic voltammogram of compounds **1**- **5** v/s Fc/Fc^{+} measured in degassed dichloromethane solvent by using tetrabutylammonium perchlorate (TBAP) as supporting electrolyte at 50 mV/s scanning rate.

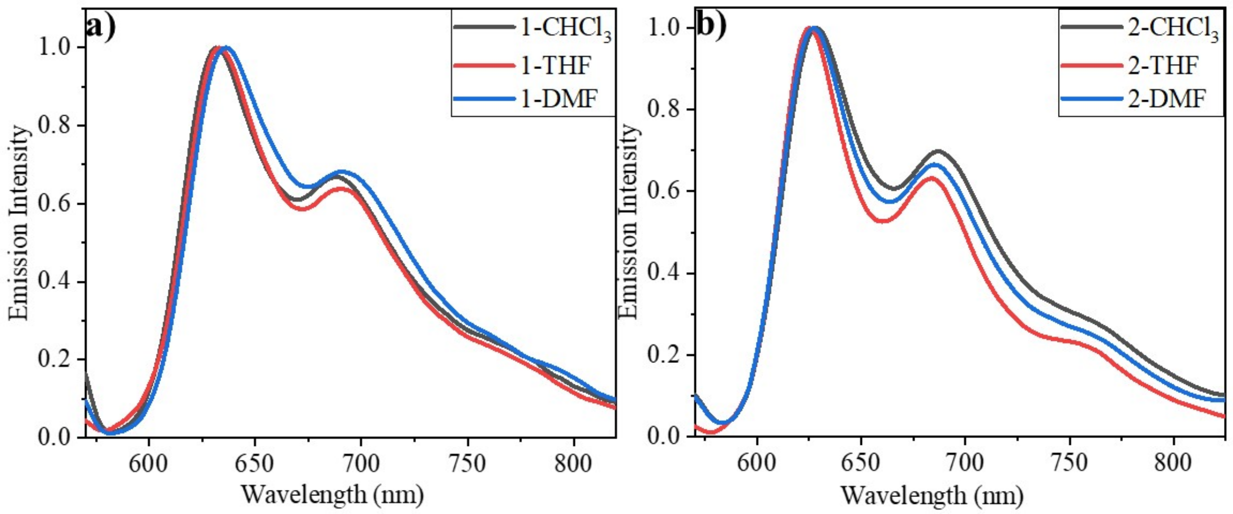

Fig. S 4: Fluorescence solvatochromism of compounds **1**-**5**.

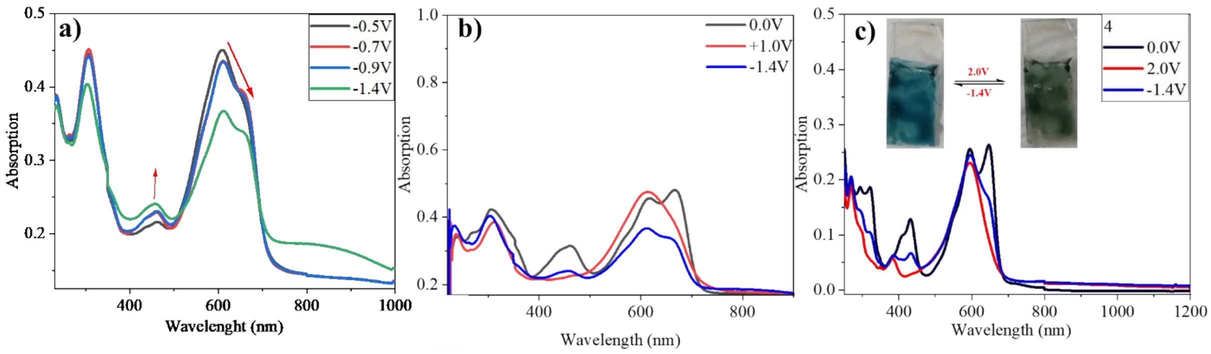

Fig. S 5: The Spectro electrochemical absorption spectroscopy of compound **5** reversibility test (**a**); the Spectro electrochemistry of **5** at 0 V, +1.0 V and -1.4 V (**b**); the Spectro electrochemistry of **4** at 0 V, +2.0 V and -1.4 V.

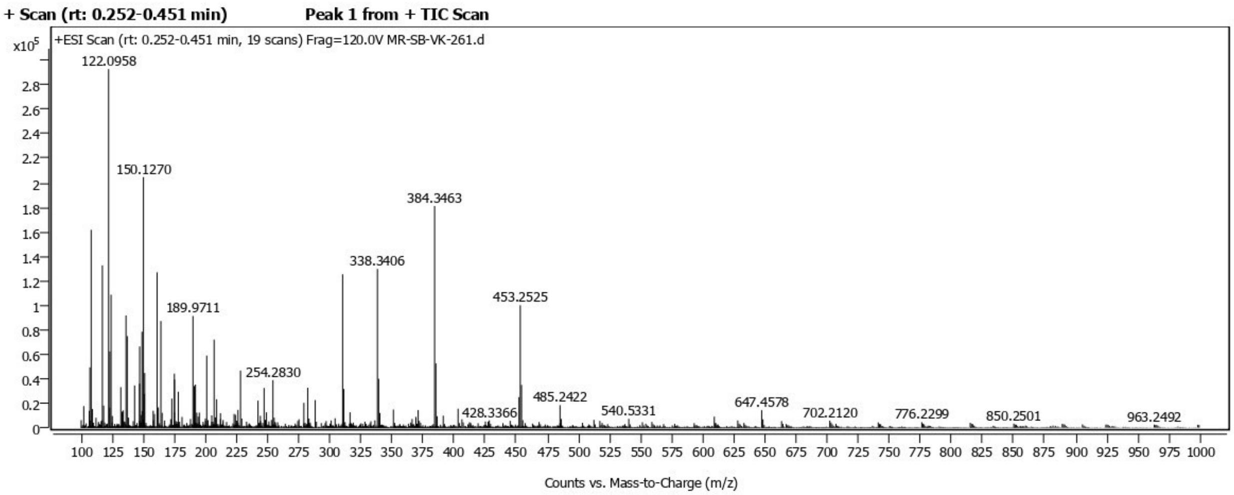

Compound Details

Cpd. 1: C30 H32 N2 O2

Formula	m/z	Observed M/Z	Difference_Da	Difference_PPM	Score
C30 H32 N2 O2	453.2525	453.252464891631	-0.782172479887322	-1.72952734936599	96.78

Compound Spectra (Zoomed)

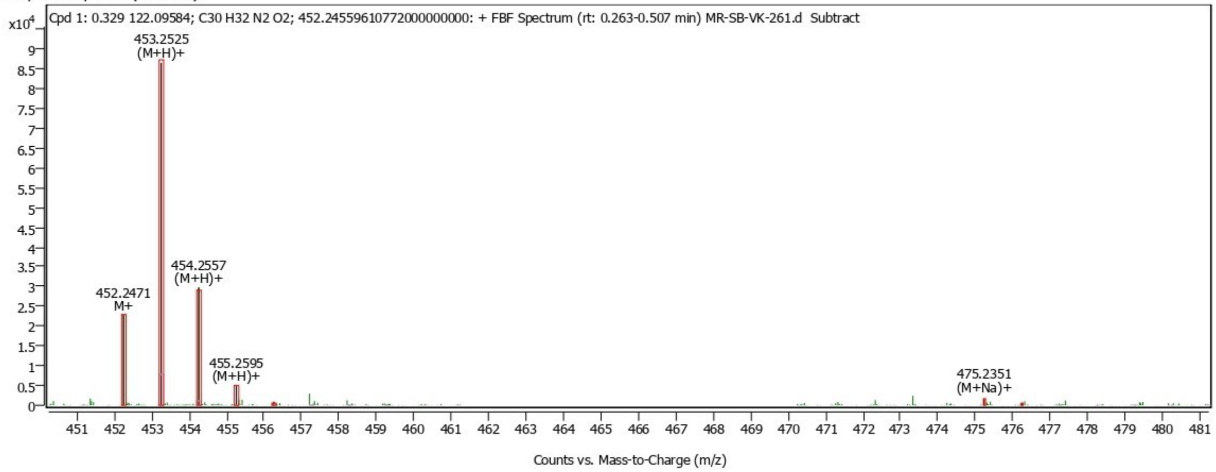

Fig. S 6: HRMS of compound **1**.

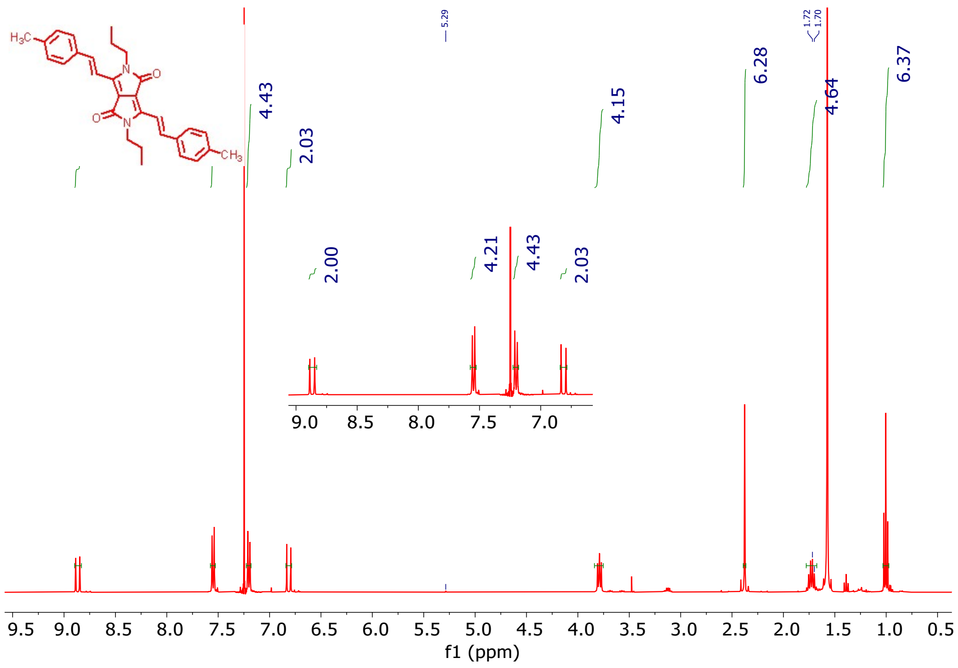

Fig. S 7: ^{1}H-NMR of compound **1**.

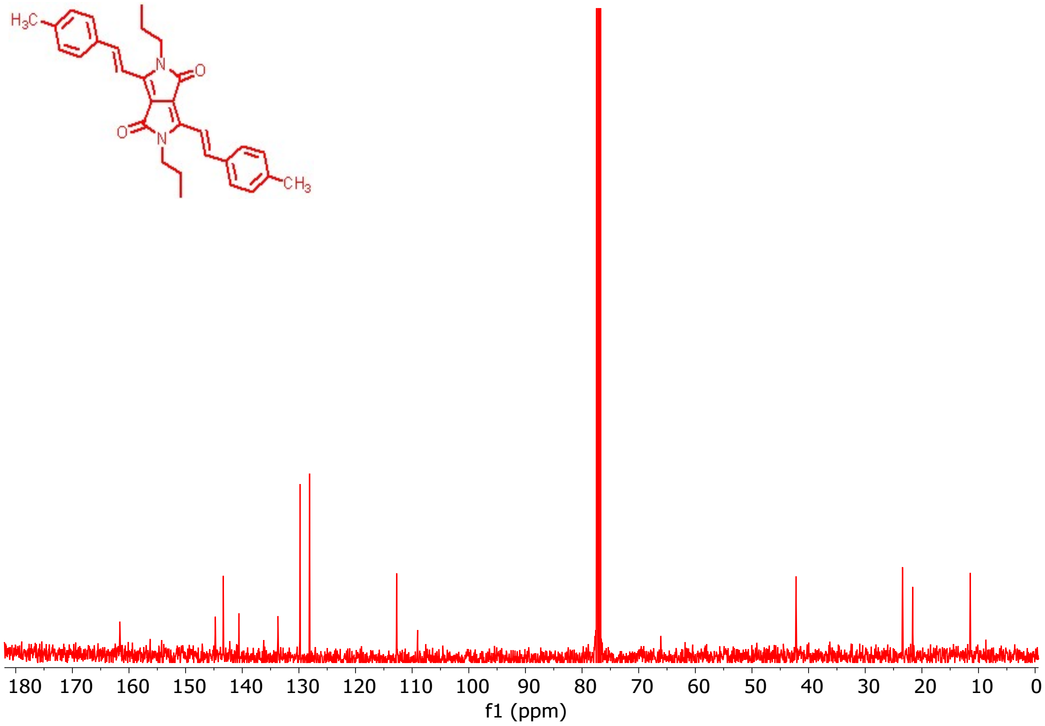

Fig. S 8: ^{13}C NMR of compound **1**.

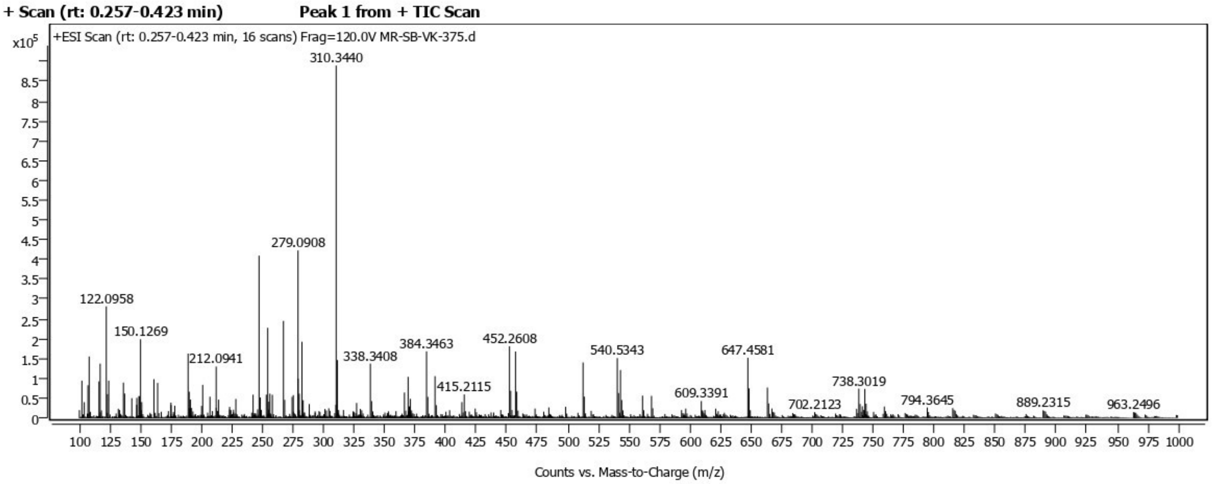

Compound Details

Cpd. 1: C30 H26 F6 N2 O2

Formula	m/z	Observed M/Z	Difference_Da	Difference_PPM	Score
C30 H26 F6 N2 O2	561.1961	561.196064964201	-0.89859071954379	-1.60408248006061	97.11

Compound Spectra (Zoomed)

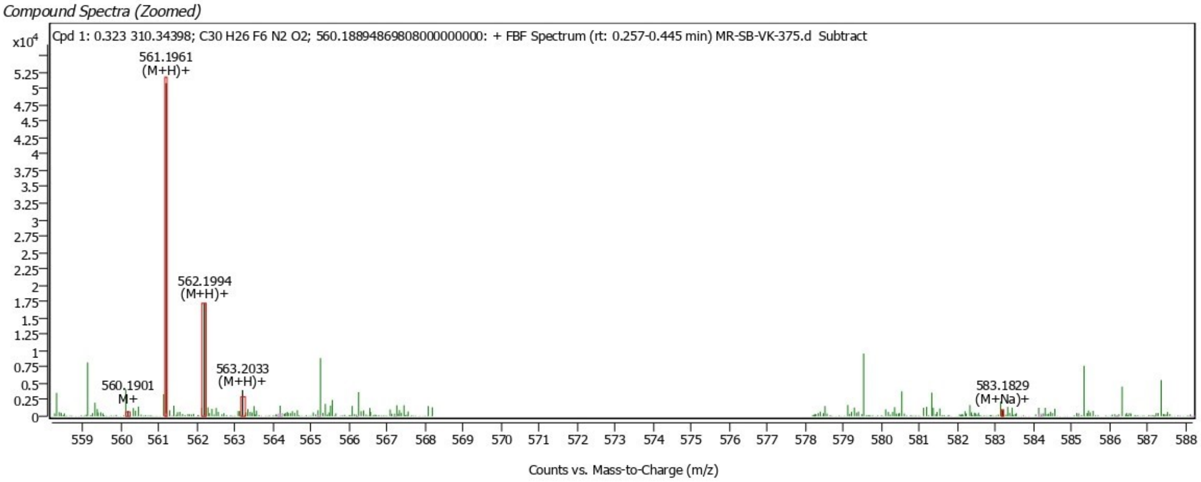

F

ig. S 9: HRMS of compound **2**.

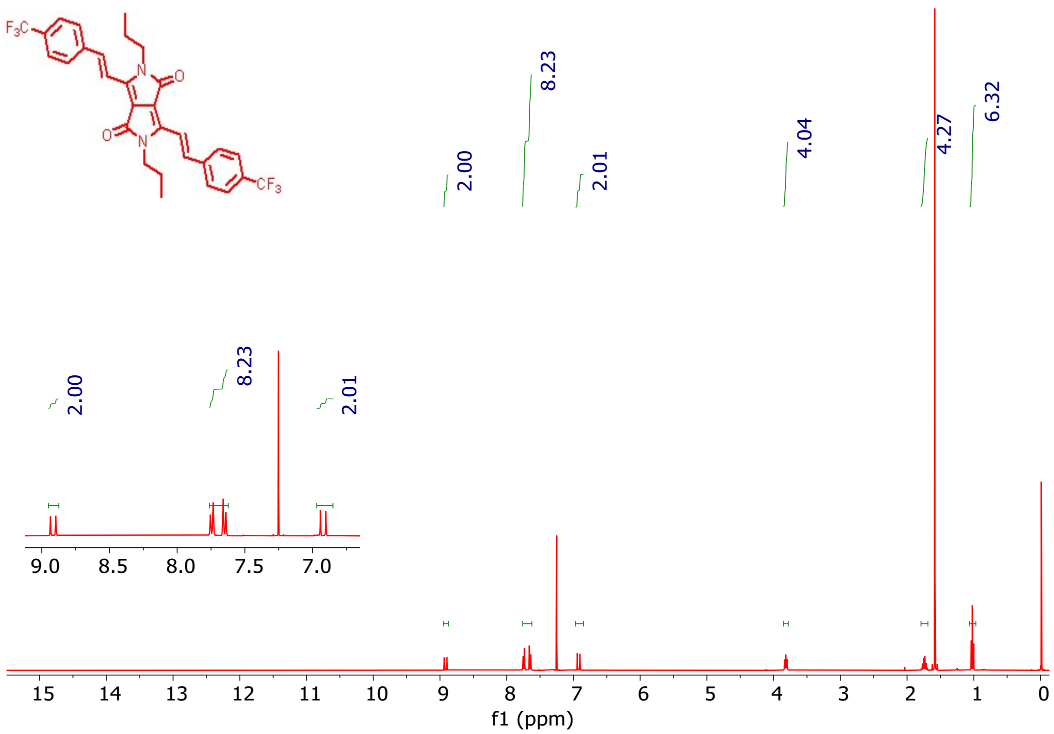

Fig. S 10: ^{1}H-NMR of compound **2.**

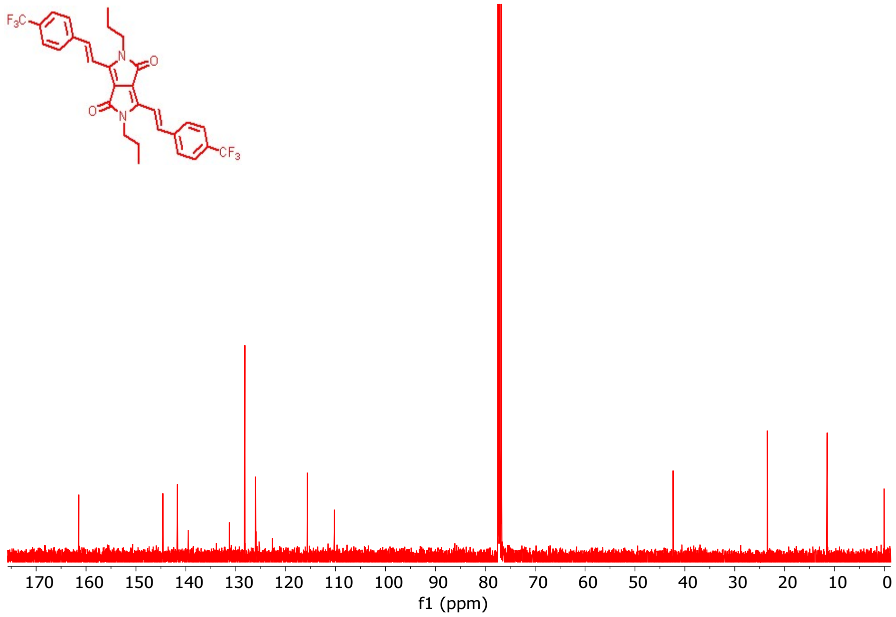

Fig. S 11: ^{13}C-NMR of compound **2**.

+ Scan (rt: 0.250-0.472 min) **Peak 1 from + TIC Scan**

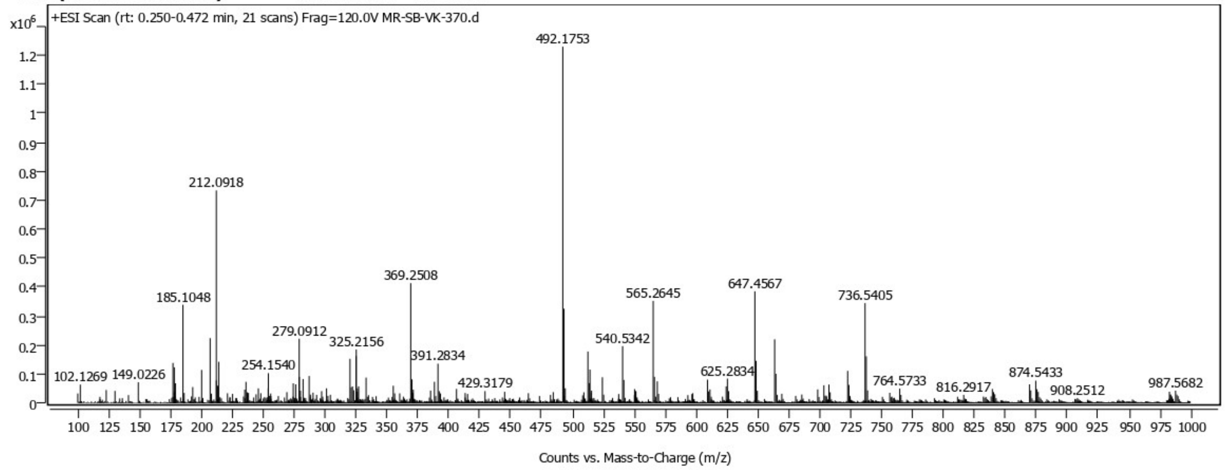

Compound Details

Cpd. 1: C44 H36 N2 O2

Formula	m/z	Observed M/Z	Difference Da	Difference PPM	Score
C44 H36 N2 O2	625.2822	625.282155542396	-1.57156676903014	-2.51741624502476	84.32

Compound Spectra (Zoomed)

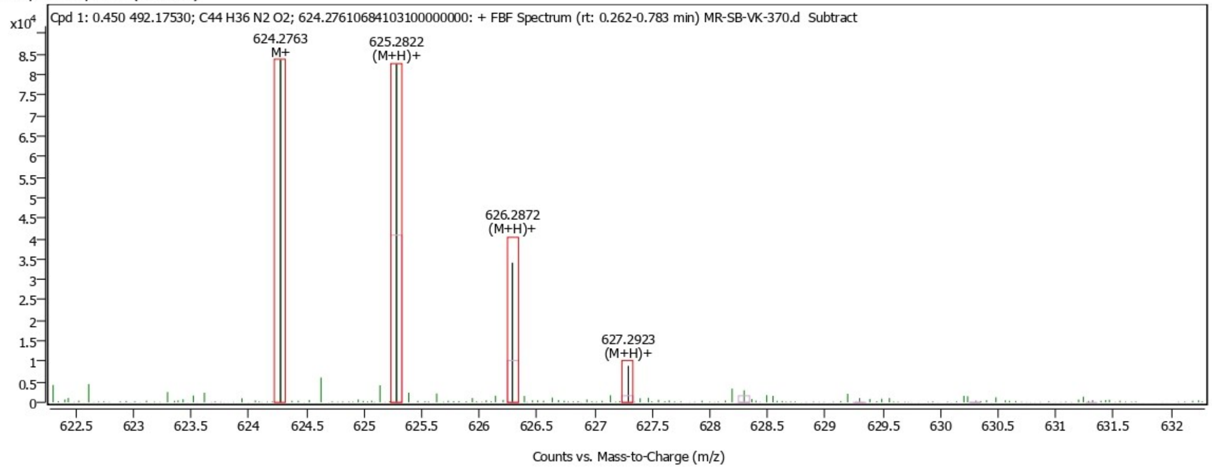

Fig. S 12: HRMS of compound **3**.

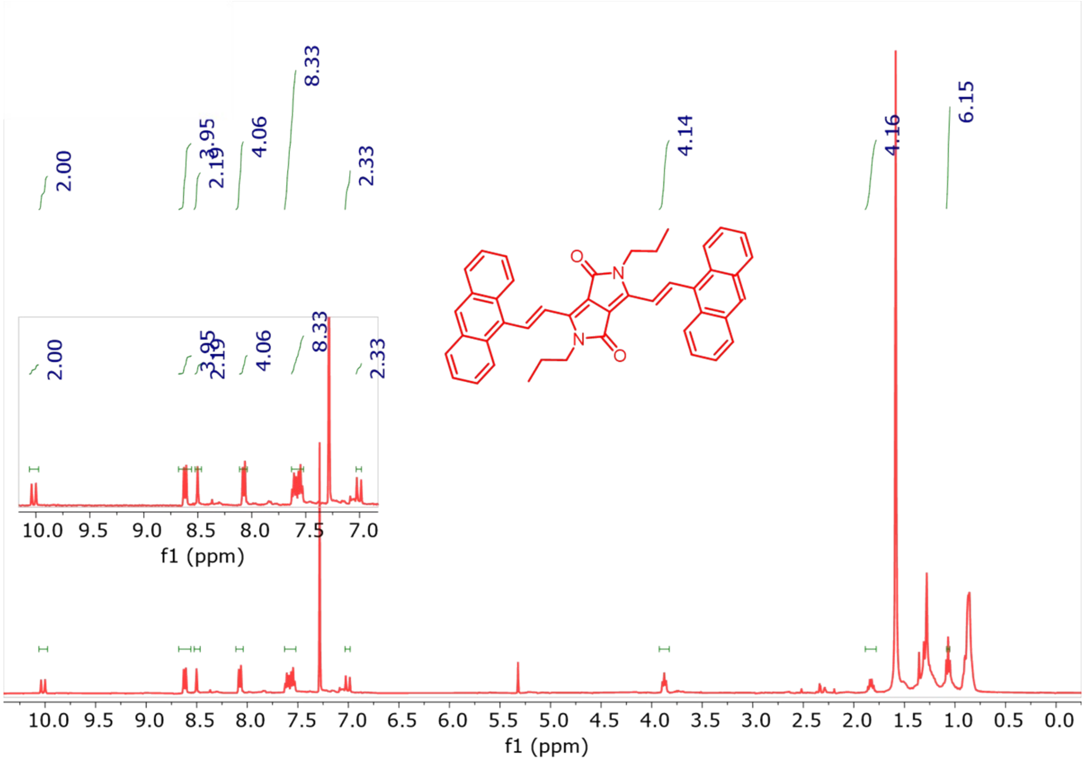

Fig. S 13: ^{1}H NMR of compound **3**.

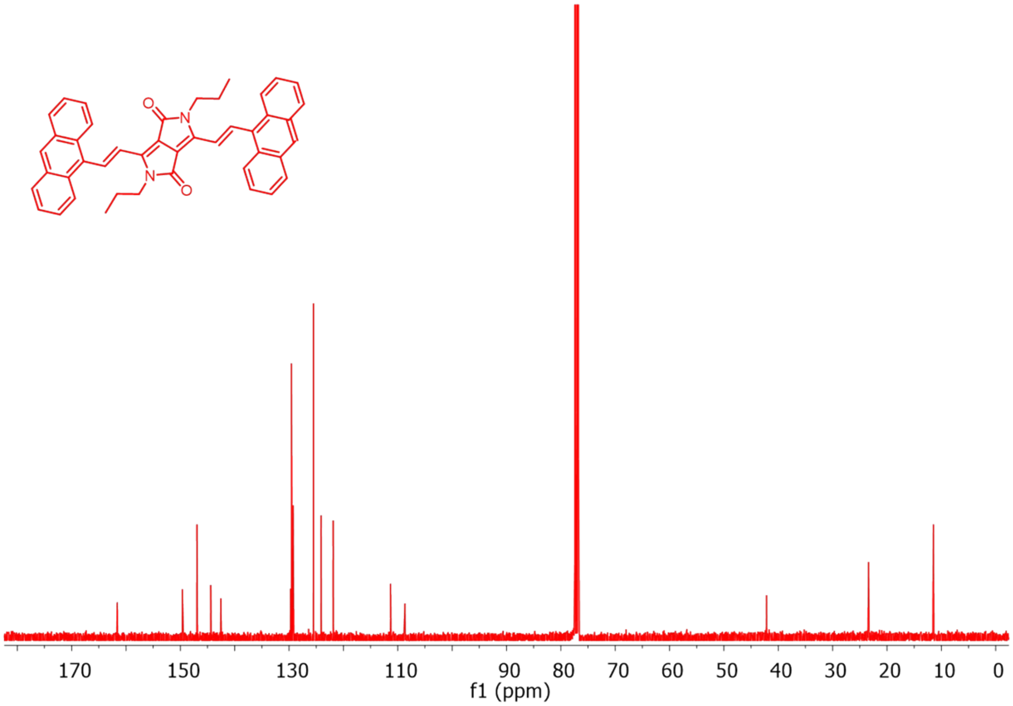

Fig. S 14: 13C-NMR of compound **3**.

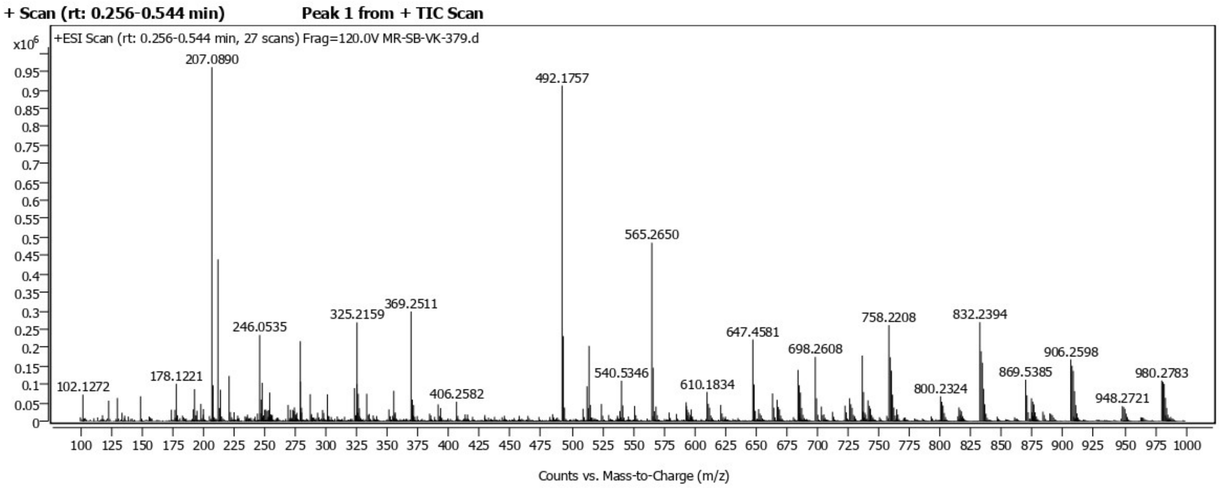

Compound Details

Cpd. 1: C52 H58 N4 O2

Formula	m/z	Observed M/Z	Difference Da	Difference PPM	Score
C52 H58 N4 O2	771.4610	771.460974673636	-0.858517153574212	-1.11429747975801	77.46

Compound Spectra (Zoomed)

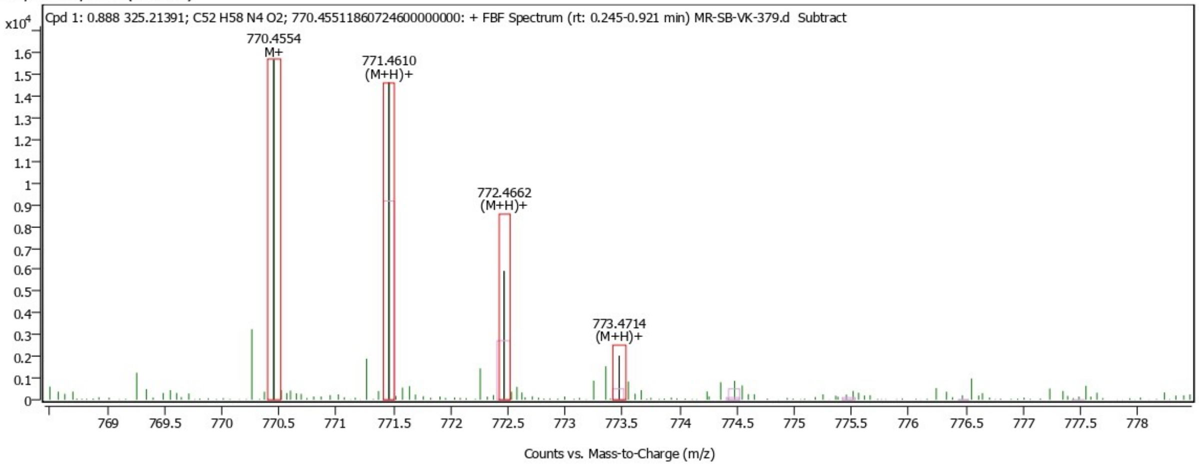

Fig. S 15: HRMS of compound **4**.

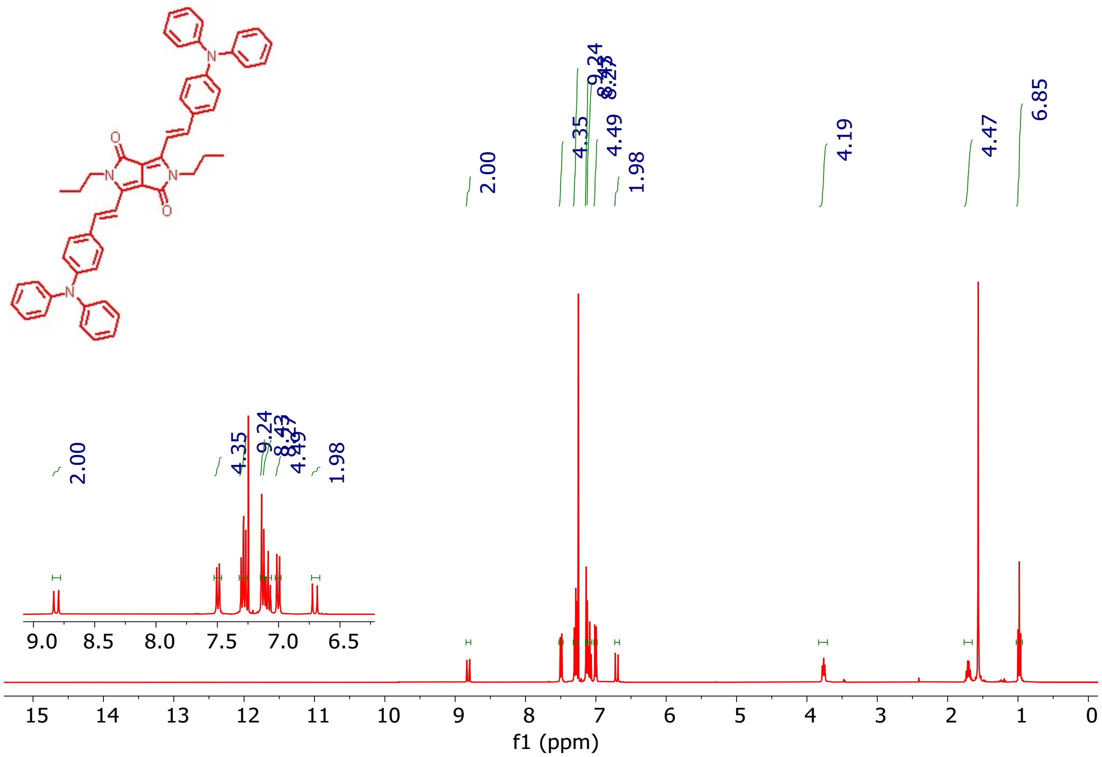

Fig. S 16: ^{1}H-NMR of compound **4.**

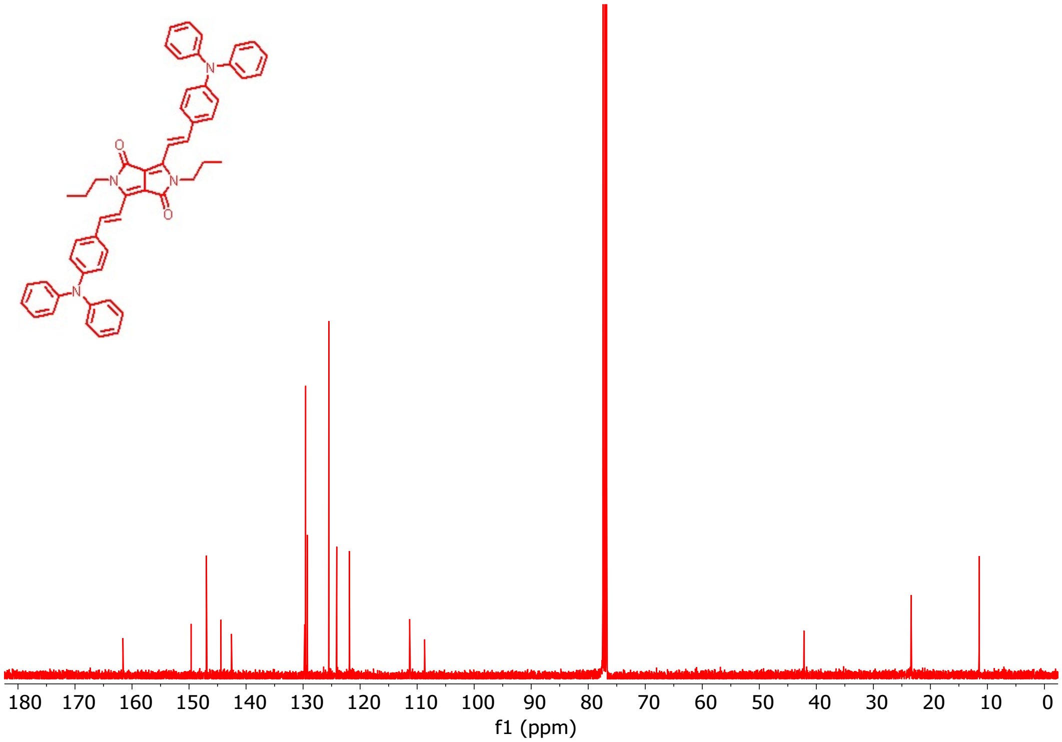

Fig. S 17: ^{13}C-NMR of compound **4.**

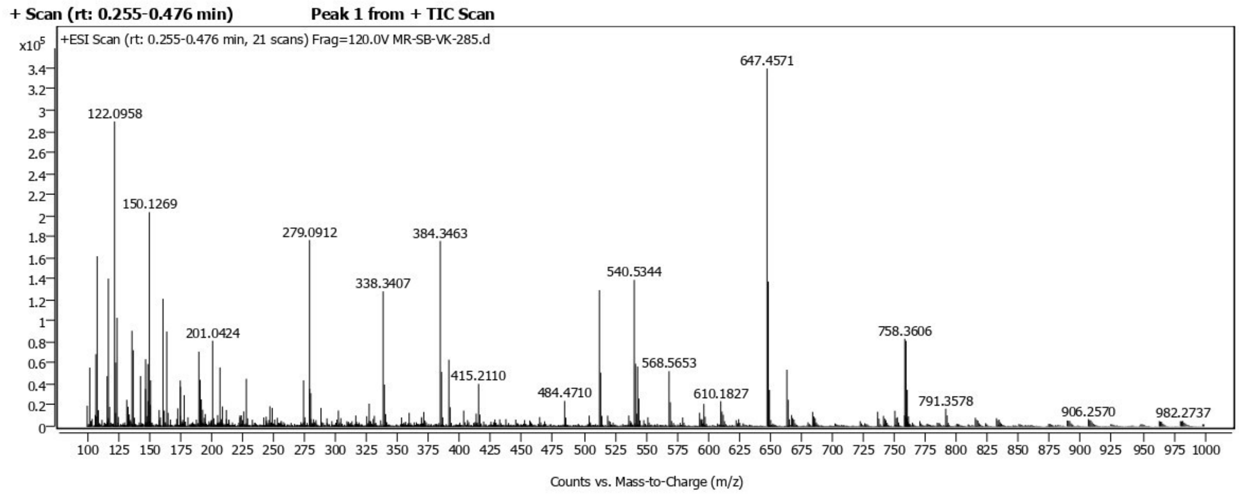

Compound Details

Cpd. 1: C52 H46 N4 O2

Formula	m/z	Observed M/Z	Difference Da	Difference PPM	Score
C52 H46 N4 O2	781.3550	781.355037212419	-0.284130977547647	-0.374664011112545	67.08

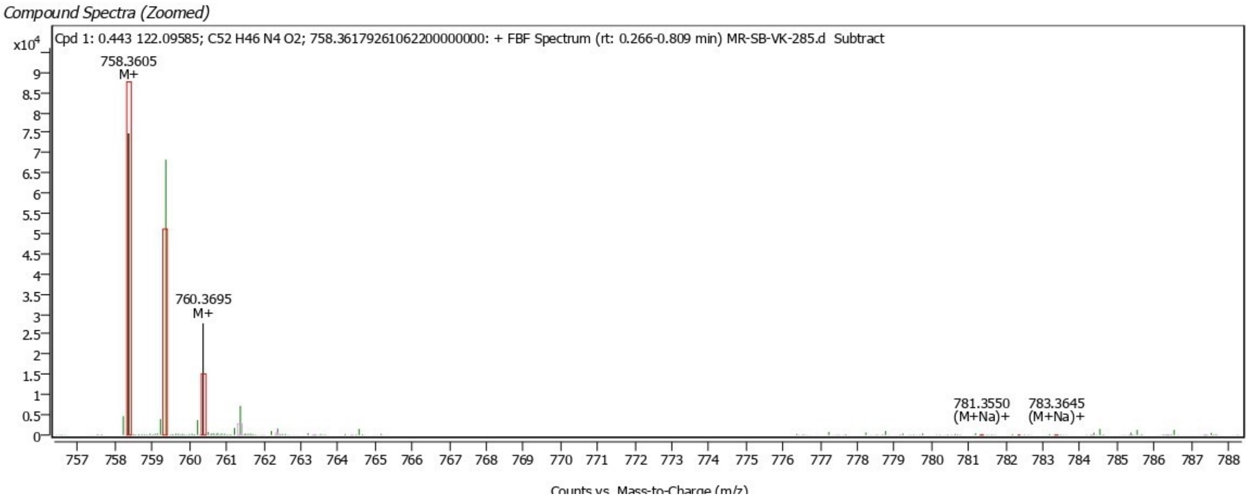

Fig. S 18: HRMS of compound **5**.

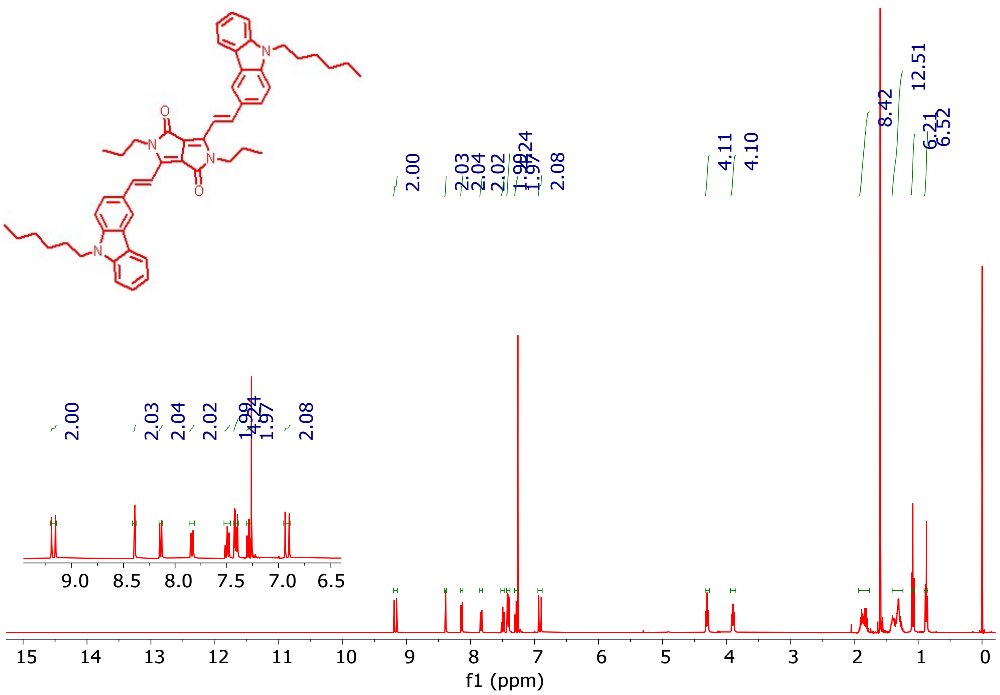

Fig. S 19: ^{1}H-NMR of compound **5.**

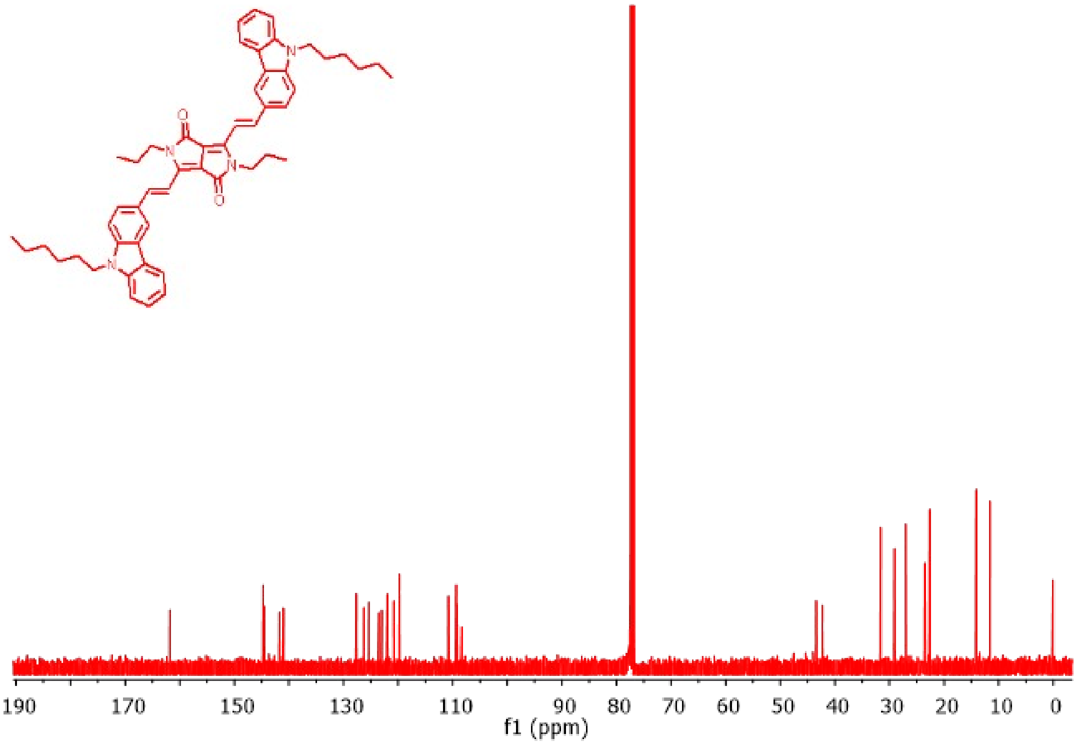

Fig. S 20: ^{13}C-NMR of compound **5**.

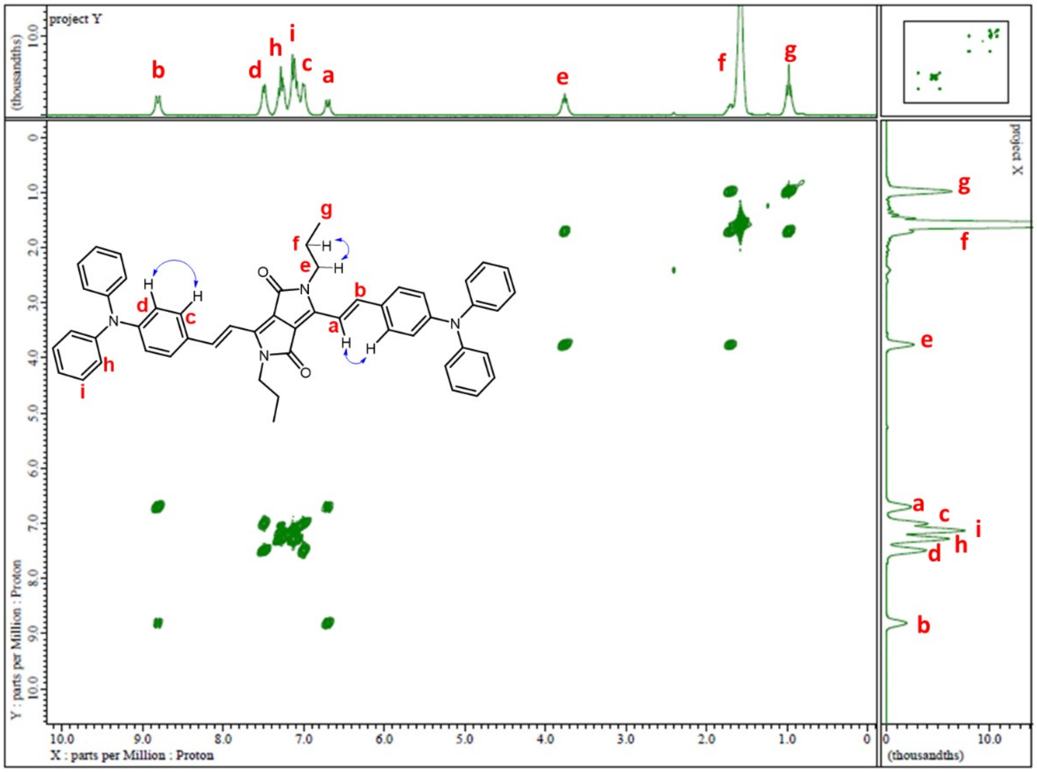

Fig. S 21: 2D COSY spectrum of compound **5**.

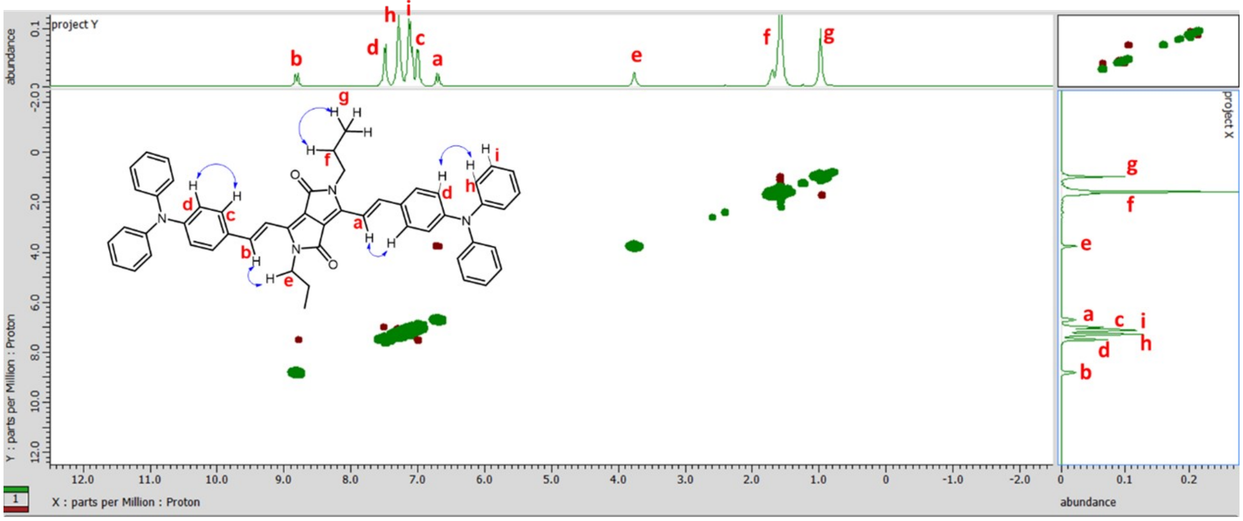

Fig. S 22: 2D NOESY spectrum of compound **5**.

3. References:

1 D. Feng, G. Barton and C. N. Scott, *Org Lett*, 2019, **21**, 1973–1978.
